# Securing Session Initiation Protocol

**DOI:** 10.3390/s22239103

**Published:** 2022-11-23

**Authors:** Osama Younes, Umar Albalawi

**Affiliations:** 1 Faculty of Computer and Information Technology, University of Tabuk, Tabuk 47512, Saudi Arabia; 2 Faculty of Computers and Information, Menoufia University, Shibin El Kom 32511, Egypt; 3 School of Computing & Data Science, Wentworth Institute of Technology, Boston, MA 02115, USA

**Keywords:** SIP security, authentication protocols, key agreement, SRP analysis, VoIP

## Abstract

The session initiation protocol (SIP) is widely used for multimedia communication as a signaling protocol for managing, establishing, maintaining, and terminating multimedia sessions among participants. However, SIP is exposed to a variety of security threats. To overcome the security flaws of SIP, it needs to support a number of security services: authentication, confidentiality, and integrity. Few solutions have been introduced in the literature to secure SIP, which can support these security services. Most of them are based on internet security standards and have many drawbacks. This work introduces a new protocol for securing SIP called secure-SIP (S-SIP). S-SIP consists of two protocols: the SIP authentication (A-SIP) protocol and the key management and protection (KP-SIP) protocol. A-SIP is a novel mutual authentication protocol. KP-SIP is used to secure SIP signaling messages and exchange session keys among entities. It provides different security services for SIP: integrity, confidentiality, and key management. A-SIP is based on the secure remote password (SRP) protocol, which is one of standard password-based authentication protocols supported by the transport layer security (TLS) standard. However, A-SIP is more secure and efficient than SRP because it covers its security flaws and weaknesses, which are illustrated and proven in this work. Through comprehensive informal and formal security analyses, we demonstrate that S-SIP is secure and can address SIP vulnerabilities. In addition, the proposed protocols were compared with many related protocols in terms of security and performance. It was found that the proposed protocols are more secure and have better performance.

## 1. Introduction

Voice over internet protocol (VoIP) reduces telecommunication costs and provides benefits to business that legacy telephone systems cannot. VoIP systems need efficient, flexible and secure transmission and signaling protocols. The real-time transport protocol (RTP) [1] is used for transmitting media streams such as voice and video over IP networks. To initiate, preserve, and stop multimedia sessions among participants, VoIP services use the session initiation protocol (SIP), which is a client/server text-based signaling protocol [2]. SIP has been used in different applications, such as video conferences, voice/video distribution, and online games [3].

Due to the rise in VoIP applications, the issue of security in SIP has become pivotal [4,5]. According to the security analysis of [6,7], the major source of attacks on VoIP technology is due to vulnerabilities of SIP. Although SIP is widely used as a signaling protocol, it is vulnerable to several attacks, such as registration hijacking, impersonation, message tampering, eavesdropping, and man-in-the-middle attacks. Such attacks degrade the trust level of users to completely rely on VoIP technologies.

To avoid most SIP threats, the SIP protocol needs to support a number of security mechanisms: authentication, confidentiality, integrity, and availability services [8,9]. Authentication mechanisms are used to prevent different masquerading and spoofing attacks. Attacks related to eavesdropping on signaling, media, or network management sessions can be avoided using confidentiality mechanisms. The lack of integrity mechanisms allows malicious users to manipulate messages to perform different attacks such as replaying, malformed message, and spoofing attacks [10]. Availability mechanisms protect the availability of entities in VoIP systems. They can prevent different denial-of-service (DoS) attacks and flooding attacks.

SIP security basically relies on well-known internet security standards that support authentication, confidentiality, integrity, and/or availability [2], which are HTTP digest [11], IP security (IPSec) [12], transport layer security (TLS) [13], and secure multipurpose internet mail extensions (S/MIME) [14]. These standard security mechanisms are applied in existing and widely used internet applications and services. However, none of these SIP security solutions are a silver bullet; each of them has many demerits, which are discussed in Section 2.

Numerous authentication and key agreement schemes have been proposed in the literature for SIP. However, most of them did not support confidentiality, integrity, and/or availability. These schemes can be divided into two groups: public key infrastructure (PKI) mechanisms and PKI-free mechanisms. PKI mechanisms [15] can effectively provide mutual authentication and key agreements over public channels. However, these approaches require users to store additional data (e.g., certificates) on their devices, which are vulnerable to physical attacks. Additionally, cryptographic operations during authentication and key exchange processes are computationally expensive. PKI-free mechanisms, which are also known as password-authenticated key exchange (PAKE) mechanisms, are considered a promising alternative solution to these concerns [16]. PAKE mechanisms do not require clients to store any secret information but rather allow them to authenticate with a server by entering the user’s password. In addition, they are computationally lightweight cryptographic protocols that make them suitable for VoIP systems.

PAKE schemes are divided into two categories: balanced PAKE (B-PAKE) and augmented PAKE (A-PAKE). In balanced PAKE schemes, to authenticate a user, the server stores a value derived from the user’s password, which is used to establish a common secret key between the client and the server. However, these schemes cannot protect the password if the server is compromised, because an attacker can use this value to impersonate the user or the server. 

A-PAKE authentication protocols can provide more security than B-PAKE protocols. Nevertheless, they are also more complex and computationally expensive to implement. Many A-PAKE-based authentication protocols designed for general client–server applications have been proposed in the literature: SRP [17], AugPAKE [18], OPAQUE [19], Augmented-EKE [20], B-SPEKE [21], J-PAKE [22], and PAKE2+ [23]. The secure remote password (SRP) protocol was introduced in [24] and is described as SRP-3 in RFC 2945 [25]. SRP-6 is a variant of SRP-3 that is more flexible and secure. As described in RFC 5054 [26], it is used for password authentication in SSL/TLS. Additionally, it is included in IEEE P1363 [27] and ISO/IEC 11770-4 standards. 

According to the Stanford SRP homepage [28], “SRP is most widely standardized protocol of its type, and as a result is being used by organizations both large and small, commercial and open-source, to secure nearly every type of human-authenticated network traffic on a variety of computing platforms”. The SRP protocol is currently at revision 6a [17,28]. Compared to other A-PAKE protocols, SRP is the more practical choice because it has more available implementations, is built in integration with TLS [26], and is faster and secure enough for common use [17]. Unfortunately, as we prove in Section 4, SRP-6a is vulnerable to offline password guessing attacks, stolen verifier attacks, and Denning–Sacco attacks. Many A-PAKE-based authentication schemes for SIP have been introduced in the literature [29,30,31,32,33,34,35,36,37,38,39,40,41,42,43,44,45,46,47,48,49,50,51,52]. However, most of these schemes have security vulnerabilities, as explained in Section 2.

To secure SIP, this work introduces a new protocol called secure-SIP (S-SIP). As explained in detail in Section 6, S-SIP thwarts most SIP attacks such as impersonation, replay, man-in-the-middle, session teardown, registration hijacking, request spoofing, message tampering, and re-invite attacks. S-SIP consists of two protocols: the SIP authentication (A-SIP) protocol and the key management and protection (KP-SIP) protocol. A-SIP is an authentication protocol based on SRP. A-SIP is more secure and efficient than SRP. It not only covers all the security flaws and weaknesses of SRP but also provides more functionalities. To stop impersonation and spoofing attacks, A-SIP is used to secure the registration of the authenticated users to the proxy server and distribute the session keys. KP-SIP is used to exchange session keys between clients and secure the signaling messages of SIP. It provides confidentiality and integrity services for SIP. To evaluate the security attributes of the proposed protocols, the ProfVerif [53] tool is used, which is one of the best tools for automated security analysis of cryptographic protocols. The main contributions of this work are as follows:

1. For the first time, we provide an informal security analysis for the SRP protocol.
2. Based on the SRP protocol, we introduce a new authentication scheme that overcomes vulnerabilities and weaknesses discovered in SRP.
3. A new protocol is introduced to provide confidentiality and integrity services for SIP.
4. The security of the proposed protocols is formally and informally analyzed.
5. The proposed protocols are compared with many related protocols in terms of security and performance.

The rest of the paper is organized as follows: The related work is discussed in Section 2. In Section 3, basic concepts about SIP and SRP protocols are explained. Section 4 presents informal security analysis of the SRP protocol. Section 5 explains the proposed protocol S-SIP. The informal and formal security analyses of S-SIP are discussed in Section 6 and Section 7, respectively. Performance analysis and comparison are presented in Section 8. Finally, some conclusions are drawn in Section 9.

## 2. Related Work

Many security mechanisms have been applied to protect SIP from various attacks. These mechanisms can be divided into two categories: standard-based and research-based solutions. These solutions are discussed in this section.

### 2.1. Standard-Based Solutions

To secure SIP, well-known internet security mechanisms were adopted. More specifically, the following internet security standards were usually used for securing SIP: HTTP digest, TLS, IPSec and S/MIME.

(1) HTTP Digest

HTTP digest [11] is a simple challenge–response protocol that uses a shared secret along with a username, a domain name, a nonce, and specific fields from the SIP message to compute a cryptographic hash, which is used for entity authentication. An SIP server or a client can challenge the request initiator to send a proof of its identity. 

Although the HTTP digest protocol can offer one-way message authentication and replay protection, it does not provide mutual authentication, message integrity or confidentiality [8]. Therefore, it cannot thwart man-in-the-middle (MITM) and impersonation attacks. Moreover, if short or weak passwords are used as a shared secret, the HTTP digest protocol becomes vulnerable to offline dictionary attacks [9].

(2) Transport Layer Security

The transport layer security protocol [13] is defined in RFC 4346. TLS provides security services at the transport layer of the internet architecture. TLS offers authentication, integrity, and confidentiality services among SIP entities. It is widely used on the internet today to secure web browsing. TLS is composed of two protocols: the TLS record protocol and the TLS handshake protocol. The TLS handshake protocol is used for mutual authentication and to negotiate cryptographic properties of the respective session. The TLS record protocol aims to maintain a secure connection between two end points. The TLS handshake has to be completed successfully before transmitting any data. SIP Secure (SIPS) protocol [54] is a standard protocol for securing SIP based on TLS. It constructs TLS secure tunnels among each hop in the path between end points.

TLS adopts PKI to ensure confidentiality of all transmitted data, and to verify and authenticate the validity of each party in the context of public and private keys. Unfortunately, PKI is vulnerable to MITM attacks [55,56]. In addition, maintaining many open TLS connections simultaneously may significantly reduce the performance of SIP servers, rendering them unable to process incoming requests due to resource exhaustion, making servers more vulnerable to denial-of-service attacks [8,10,57,58]. Moreover, as we prove in Section 4, the standard password-based authentication protocol (SRP) supported by TLS (SRP-TLS) [26] is vulnerable to offline password guessing attacks, stolen verifier attacks, and Denning–Sacco attacks. The proposed protocol (S-SIP) covers security flaws and weaknesses in SIPS. S-SIP does not depend on TLS. However, it is a redesign for SIP taking into account its security requirements. In addition, the performance of the proposed protocol is better than SIPS, as explained in Section 8.

(3) S/MIME

S/MIME [14] is a standard defined in RFC 3851. Multipurpose internet mail extensions (MIME) and its security improvement S/MIME were originally designed for e-mail services. Additionally, they are used by other text-based internet application protocols. S/MIME is the de facto standard for securing emails by using a PKI infrastructure and X.509 certificates. S/MIME supports the exchange of complex messages along with preserving a set of security objectives, including confidentiality, integrity, and authenticity. S/MIME provides end-to-end confidentiality for SIP messages, integrity protection for the body and identity authentication for the sender of the message.

S/MIME has many limitations and drawbacks [57,59]. The complexity required to implement S/MIME to protect SIP signaling messages can be a significant factor in limiting its implementation in most environments. In addition, S/MIME encapsulates SIP messages into the MIME body, which creates a considerable overhead and processing cost over SIP messages. Moreover, due to the complexity of managing and distributing security certificates, most commercial SIP clients do not support S/MIME.

(4) IPSec

IPSec [12] is an independent and general purpose network-level protocol in the internet architecture designed by the Internet Engineering Task Force (IETF), which provides protection to IP packets. Thus, protocols such as SIP and RTP can run over it without any changes. IPSec can be used in a tunnel or transport mode to protect its payload. It can provide confidentiality, integrity, and authentication services for media and SIP signal messages by creating secure tunnels between end points. However, it has many drawbacks [8,9,10,57]:

- Since IPSec is implemented at the operating system level or kernel layer, many SIP clients do not support it. Thus, IPSec can be utilized to protect signaling traffic between SIP servers but not between servers and clients.
- For applications working on top of IPSec, such as SIP, it is difficult to detect whether IPSec has failed to be set up. As a result, there are advantages for security mechanisms working at layers above the IP layer.
- Deploying IPSec in VoIP systems requires more effort because of its complexity and infrastructure requirements compared to other protocols.
- It does not scale well for large, distributed networks and distributed applications.

### 2.2. Research-Based Solutions

Numerous security schemes have been introduced in the literature for securing SIP. However, most of them are only concerned with SIP authentication. The following discusses the most significant SIP authentication schemes.

In [29], Yang et al. proposed an authentication scheme based on the Diffie–Hellman key exchange protocol. Later, Huang et al. [30] identified that Yang et al.’s protocol was insecure against offline password guessing attacks. Therefore, they presented an improved scheme. Later, it was demonstrated that Huang et al.’s scheme could not thwart offline password guessing attacks [31].

Based on Yang et al.’s study, in [32,33] authentication schemes for SIP were proposed based on ECC [34]. However, Yoon et al. [35] showed that these schemes were susceptible to offline password guessing, Denning–Sacco, and stolen verifier attacks. To overcome these weaknesses, Yoon et al. proposed an enhanced authentication scheme for SIP with more security. Unfortunately, Pu [36] showed that the scheme of Yoon et al. was still prone to offline password guessing and replay attacks.

To reduce the high computational cost, Tsai [37] suggested an efficient authenticated key agreement scheme adopting only one-way hash functions and exclusive-OR operations. Nevertheless, in [38] it was proven that Tsai’s scheme could not provide perfect forward secrecy and was vulnerable to offline password guessing attacks, Denning–Sacco attacks, and stolen-verifier attacks. Thus, Yoon et al. [38] proposed an enhanced scheme to overcome the shortcomings of Tsai’s scheme. The proposed scheme was based on the elliptic curve discrete logarithm problem (ECDLP). However, Xie [39] demonstrated that Yoon et al.’s scheme could not resist offline password guessing and stolen-verifier attacks. Then, he proposed an improved scheme to overcome the weaknesses of the scheme introduced in [38]. Nevertheless, Farash and Attari [40] demonstrated that the scheme introduced in [39] was still insecure and vulnerable to offline password guessing and impersonation attacks. To avoid these vulnerabilities in Xie’s scheme, they presented an improved scheme. Based on the work introduced in [40], Zhang et al. [41] proposed an authentication scheme for SIP.

Lu et al. [42] found that Zhang et al.’s scheme was insecure against insider attacks and could not provide proper security. To overcome the weaknesses of Zhang et al.’s scheme, Lu et al. proposed an improved authentication scheme. They demonstrated that their scheme was resistant to possible known attacks. In [43] the authors stated that Lu et al.’s scheme was prone to user and server impersonation attacks. Therefore, they proposed an enhanced scheme that resists these attacks. Nevertheless, the authors in [44] found that the work introduced in [43] was still vulnerable to the attacks that appeared in Lu et al.’s scheme. Meanwhile, they indicated that Lu et al.’s [42] scheme was insecure against impersonation and identity guessing attacks.

In [45], to overcome vulnerabilities in previous schemes, Zhang et al. developed an authentication and key agreement scheme for SIP using a smart card. In addition to the password, the smart card is used as a second authentication factor. The authors showed that their scheme was efficient and secure against several attacks. Irshad et al. [46] proved that Zhang et al.’s scheme could suffer from impersonation attacks. Consequently, they suggested an improvement for Zhang et al.’s authentication scheme. However, Arshad and Nikooghadam [47] proved that the scheme introduced in [46] could not prevent user impersonation attacks.

In [48], Tu et al. developed an authentication scheme that had low computational costs, based on Zhang’s scheme [45]. Chaudhry et al. [49] showed that Tu et al.’s scheme [48] was not immune to server impersonation attacks and replay attacks. Thus, they developed a lightweight authentication and key agreement protocol for SIP. However, Nikooghadam et al. [50] performed cryptanalysis on Chaudhry et al.’s scheme and showed its weakness against password guessing attacks. They developed an enhanced scheme to overcome this weakness. Later, Ravanbakhsh et al. [51] demonstrated that the work introduced in [50] was insecure because it did not provide perfect forward secrecy. Thus, they presented a two-factor authentication and key agreement scheme for SIP networks and showed that it was resistant against various active and passive attacks. Nevertheless, it was proven that Ravanbakhsh et al.’s scheme [51] did not provide perfect forward secrecy [52].

Few studies have been introduced in the literature that provide confidentiality, integrity, and authenticity services for VoIP systems. The most recent and significant studies are discussed in the following. In [60], the authors studied security threats and attacks in internet protocol multimedia subsystem (IMS) networks. In addition, they presented a framework model for protecting messages exchanged between users and for user authentication using IPsec and HTTP digest protocols on every single gateway router. HTTP digest provides user authentication and a limited degree of integrity protection. The IPsec protocol protects confidentiality and integrity at the network layer. However, as explained in Section 2.1, HTTP digest and IPsec protocols have many drawbacks.

In [61], Farley et al. presented a system called VoIP Shield to mitigate MITM and replay attacks. The VoIP Shield consists of two shields with a pre-distributed shared key; one of the shields is connected to the client and the other is connected to the proxy server to protect VoIP traffic exchanged between them. For message authentication, the shields use a simple hash-based message authentication code scheme. However, the VoIP Shield did not support any scheme for entity authentication or key distribution and management which made it vulnerable to many VoIP attacks. 

Basem et al. [62] provided a multilayered security solution based on different open-source applications: Snort, IPtables, SnortSam, and OpenVPN tunnel. The design provided reliable and secure VoIP services for users to prevent denial-of-service and eavesdropping attacks. Signaling messages were protected using the OpenVPN tool which transmits traffic over TLS-based VPNs. The Snort tool was used for detecting security violations and attacks. However, as explained in Section 2.1, the TLS protocol has many drawbacks. In addition, intrusion detection and prevention tools or techniques affect the system’s performance.

## 3. Background

### 3.1. Session Initiation Protocol

SIP has been standardized by the IETF [2]. It is an application layer signaling protocol for setting up, modifying and terminating multimedia IP sessions, including VoIP telephony, video, streaming media, and instant messaging. SIP is a text-based protocol based on the HTTP protocol, which defines two types of messages: SIP requests and responses. The SIP protocol follows the client/server model whose basic components are [9]:

(1) User Agent Client and Server:

An SIP user agent is a logical network endpoint used to create or receive SIP messages and thereby manage an SIP session. The user agent itself has a client element, called the user agent client (UAC), and a server element, called the user agent server (UAS). The UAC is responsible for creating requests and the UAS processes and responds to each request generated by a UAC. The SIP user agent (UA) can be lightweight clients suitable for embedding into end-user devices such as mobile handsets; alternatively, they can be desktop applications (e.g., softphones).

(2) Registrar Server

The SIP registrar server is responsible for user registration. It has a database containing the location and user’s preferences as indicated by the user agents. The registrar server accepts SIP registration requests and binds the information it receives (the SIP address and associated IP address of the registering device).

(3) Proxy Server

The proxy server is an intermediary entity that acts as both a server and a client for the purpose of making requests on behalf of other clients. A proxy server primarily plays the role of routing. Its job is to ensure that a request is sent to another entity (proxy server) closer to the targeted user agent. Users in an SIP environment are identified by SIP uniform resource identifiers (URIs). The format of an SIP URI is similar to an e-mail address, generally consisting of a username and a domain name. 

**Figure 1:**
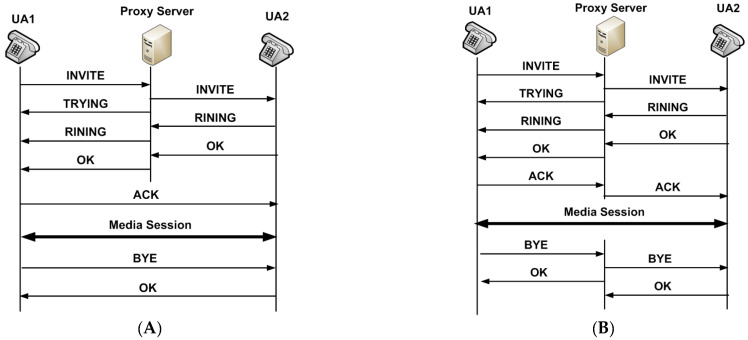
An example of a SIP call: (**A**) without the record-route option; (**B**) with the record-route option.

SIP is designed to be simple and easy to use. It defines six main types of messages, namely INVITE, ACK, BYE, CANCEL, OPTIONS, and REGISTER. Figure 1 shows an example of a call flow from one UA (UA1) to another (UA2) [2]. A session is initiated when UA1 sends an INVITE message to the appropriate proxy server indicating that UA1 wishes to communicate/talk with UA2 [9]. Immediately, the proxy server sends a response message (TRYING) to UA1 to acknowledge that the INVITE message is handled and attempts to resolve the called user’s location and sends the request to UA2. UA2 sends a response (RINGING) when their telephone begins to ring. Finally, when UA2 receives the request and picks the call, it sends the OK message to the proxy server that forwards it to UA1. Upon receiving the OK message, UA1 sends the ACK message to UA2. Upon reception of the ACK message, UA2 establishes the connection with UA1. Then, media streams are exchanged directly between them. 

The session ends with a BYE request message which is routed directly from UA1 to UA2 that sends a reply with an OK message, as shown in Figure 1A. In some cases, it may be useful for proxy servers in the SIP signaling path to see all messages exchanged between the endpoints for the duration of the session. In these cases, the proxy servers insert a record-route field in the INVITE and OK messages, which enforces the BYE and OK messages to be routed through the proxy servers [2], as shown in Figure 1B. If there are more than one proxy server between UA1 and UA2, all SIP messages received from user agents are routed through the proxy servers.

### 3.2. Secure Remote Password Protocol

The SRP protocol is an A-PAKE protocol proposed in [24]. It is also known as SRP-3 and described in RFC 2945 [25]. SRP-6 [28] is a variant of SRP-3 that is included in IEEE P1363 [27] and ISO/IEC 11770-4 standards. Additionally, it is described in RFC 5054 for strong password authentication in SSL/TLS [27]. The SRP protocol is currently at revision 6a [17,28].

SRP is suitable for secure password verification and session key generation over an insecure communication channel. The protocol allows the participants to establish secure sessions without actual exchange of a password or any other information that can be derived from the password. Furthermore, being an augmented PAKE protocol, the server does not store any password-equivalent data. Therefore, the SRP protocol allows a client and a server to authenticate each other without transmitting a password or trusting a third party.

**Figure 2:**
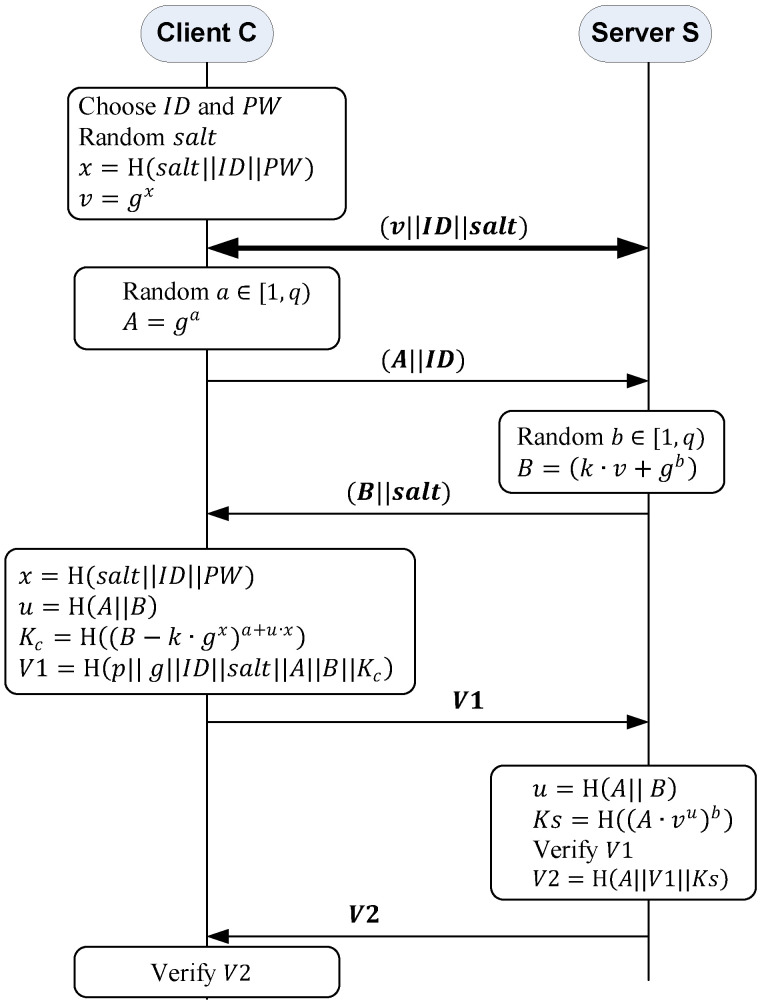
The secure remote password protocol SRP-6a.

The main goal of SRP is to establish a key agreement between two parties in a client/server model to authenticate themselves in a manner similar to Diffie–Hellman key exchange. Figure 2 shows the operation of the SRP-6a protocol between client *C* and server S. Additionally, Table 1 shows the notation used in this section and Section 4 and Section 5. In SRP, all computations are performed in a finite Galois Field GF(p) defined by a large prime p and a base element g that generates a large multiplicative subgroup of order q. The prime number p must be large enough so that computing discrete logarithms modulo p is infeasible. In addition, all computations are performed modulo p. This means that $g^{x}$ should be read as $g^{x}\;mod\;p$.

The SRP protocol consists of three phases: registration phase, login phase, and authentication phase. In the registration phase, the user of a client *C* must register their password with the server S. The user freely chooses their identity ID and a password PW. Then, *C* generates a random password salt and computes a verifier v and a secret exponent x, as shown in Figure 2. Then, *C* sends v, ID, and salt to S through a pre-established secure channel. S stores v and salt in its database indexed by ID.

In the login phase, the user tries to login into S over an insecure channel. *C* generates a random number a∈[1, q) and computes the ephemeral public key $A=g^{a}$. Then, *C* sends ID and A to S. Upon receiving the message, using the client’s ID, S retrieves the corresponding *salt* and the verifier v. Then, it generates a random number b∈[1, q) and computes the ephemeral public key $B=k\cdot v+g^{b}$. Next, it sends back *B* and *salt* to C. Upon receiving the message, *C* computes x, u, $K_{c}$, and V1, as shown in Figure 2. u is a parameter that two parties can compute using the public outputs: A and B. $K_{c}$ is the client key that is simplified as:

$$K_{c}=H\left({\left(B-k\cdot g^{x}\right)}^{a+u\cdot x}\right)=H\left({\left(k\cdot v+g^{b}-k\cdot g^{x}\right)}^{a+u\cdot x}\right)=H\left(g^{b\left(a+u\cdot x\right)}\right)$$

Additionally, S can compute their session key $K_{S}$ as:

$$K_{S}=H\left({\left(A\cdot v^{u}\right)}^{b}\right)=H\left({\left(g^{a}\cdot g^{x\cdot u}\right)}^{b}\right)=H\left(g^{b\left(a+u\cdot x\right)}\right)$$

Thus, at the end of the second phase, the client and server establish a common session key $K_{SC}=K_{S}=K_{c}$.

In the last phase, the authentication phase, the two parties prove to each other that their keys are identical using two verification massages, V1 and V2. First, *C* computes V1 and sends it to S, as shown in Figure 2. *S* receives V1 and computes $V1^{*}$ as:

$$V1^{*}=H\left(p\left|\left|g\right|\right|ID\left|\left|salt\right|\right|A\left|\left|B\right|\right|\;K_{S}\right)$$

S checks if $V1=V1^{*}$. If so, this means that $K_{s}=K_{c}.$ Consequently, *S* computes V2 and sends it to C, as shown in Figure 2. After receiving V2, *C* computes $V2^{*}=H\left(A\left|\left|V1\right|\right|K_{C}\right)$. If V2 and $V2^{*}$ match, the session key is verified and S and *C* authenticate each other. If the verification of V1 or V2 fails, either *C* or S terminates the authentication process.

## 4. Security Analysis of SRP

The SRP-6a protocol does not have a proof model for security. Recently, a formal analysis of SRP was introduced in [63]. The authors analyzed SRP-3 using a cryptographic protocol shape analyzer (CPSA). They found that the structure of the protocol did not have any major weaknesses such as leakage of secret keys. In addition, they indicated that SRP could not resist malicious server attacks if the verifier v and the random number b were revealed (not only v). However, they did not explain how to perform this attack. In this section, we analyze SRP-6a informally. We found that it is vulnerable to offline password guessing, Denning–Sacco, and stolen-verifier attacks, as explained below in detail.

### 4.1. Offline Password Guessing Attack

In remote user authentication schemes, the user is allowed to choose their password. The user tends to choose a password that can be easily remembered for their convenience. Therefore, most passwords have low entropy so they are vulnerable to password guessing attacks. In this attack, an attacker intercepts authentication messages and stores them locally. Then, they attempt to use a guessed password to verify the correctness of their guess using these authentication messages. The SRP protocol is vulnerable to an offline password guessing attack. An attacker can perform this attack for SRP-6a, shown in Figure 2, as follows:

1. Assuming that the attacker masquerades as a fake server and persuades *C* to make an authentication attempt.
2. *C* computes the ephemeral public key A and sends it with its *ID* to the attacker.
3. To capture salt of C, the attacker starts an authentication session with the actual server S by sending A and ID, received from *C*, to *S*.
4. S computes B and sends it to the attacker with the *salt* of client *C*.
5. The attacker generates a random number b, guesses any password $PW^{*}$, and computes their own parameter $x^{*}=H\left(salt|\left|ID\right||PW^{*}\right)$ and exponential residue $B^{*}=k\cdot g^{x^{*}}+g^{b}$. Then, they send $B^{*}$ and salt to the client C.
6. The client *C* computes the message $V_{1}=H\left(p\left|\left|g\right|\right|ID\left|\left|salt\right|\right|A\left|\left|B^{*}\right|\right|K_{c}\right)$ and sends it to the attacker, where $K_{c}=H\left({\left(B^{*}-K\cdot g^{x}\right)}^{a+u^{*}\cdot x}\right)$ and $u^{*}=H\left(A||B^{*}\right)$.
7. The attacker imitates network failure or informs *C* that the password is not correct.
8. The long-term private password PW can be guessed by performing the following offline password guessing attack:
  (i) The attacker computes $x^{*}=H\left(salt\left|\left|ID\right|\right|PW^{*}\right)$, $B^{*}=k\cdot g^{x^{*}}+g^{b}$, $u^{*}=H\left(A||B^{*}\right)$, and $v=g^{x^{*}}$.
  (ii) The attacker computes a modified server key as $K_{S}^{*}=H\left({\left(A\cdot v^{u^{*}}\right)}^{b}\right)=\left({\left(A\cdot g^{x^{*}\cdot u^{*}}\right)}^{b}\right)$.
  (iii) The attacker computes a modified message $V_{1}^{*}=\left(p\left|\left|g\right|\right|ID\left|\left|salt\right|\right|A\left|\left|B^{*}\right|\right|K_{S}^{*}\right)$ and checks if $V_{1}=V_{1}^{*}$.
  (iv) If it holds, the attacker has guessed the correct secret password $PW^{*}=PW$.
  (v) If it is not correct, the attacker chooses another password from a dictionary and repeatedly performs the above verification process starting from step *i*.

Compromising the user’s secret password PW enables the attacker to impersonate the client or the server.

### 4.2. Stolen-Verifier Attack

In most existing password-based authentication schemes, servers are always the prime targets of adversaries because the users’ verifiers (e.g., passwords) are stored in the server’s database. In a stolen-verifier attack [64], an adversary who steals a password verifier from the server can use it to impersonate a legitimate user. In fact, an adversary who obtains a password verifier may further mount a password guessing attack. 

In the SRP protocol, if the password verifier v, ID and salt of a user stored in the server have been eavesdropped, the attacker can use v to masquerade as the original server S and can obtain sensitive user information. Additionally, they can easily perform offline password guessing attacks to extract the user’s password, which is used to impersonate the client. An offline password guessing attack can be performed as follows:

1. The attacker chooses a secret password $PW^{*}$ from the password dictionary.
2. The attacker computes $x^{*}=H\left(salt\left|\left|ID\right|\right|PW^{*}\right)$ and $v^{*}=g^{x^{*}}$ and checks if $v^{*}=v$.
3. If it holds, the attacker has guessed the correct secret password $PW^{*}=PW$.
4. If it is not correct, the attacker chooses another password from the password dictionary and repeatedly performs the above verification process.

Compromising the user’s secret password PW enables the attacker to impersonate the client or the server. In addition, using the stolen password verifier v of client C, the attacker executes the server spoofing attack as follows:

1. An active adversary AD may eavesdrop the communication flows between *C* and S or persuade *C* to make an authentication attempt with his server.
2. When legal client *C* wants to login into server *S*, they send the request message (A||ID) to AD.
3. AD generates a random number b and computes the ephemeral public key $B=k\cdot v+g^{b}$ using the stolen-verifier v for *C*.
4. AD sends B along with salt to C.
5. *C* computes his key $K_{c}=g^{a\cdot b+b\cdot u\cdot x}$ and the verification message V1, as explained in Section 3.2. Then, *C* sends V1 to AD.
6. Upon receiving V1 from C, AD computes u=H(A||B) and the verification message $V2=H\left(A\left|\left|V1\right|\right|K_{A}\right)$, where $K_{A}=H\left({\left(A\cdot v^{u}\right)}^{b}\right)=g^{a\cdot b+b\cdot u\cdot x}=K_{c}$, and sends it back to *C* as evidence that they have the correct session key.
7. *C* verifies V2 as explained in Section 3.2. Because $K_{A}=K_{c}$, *C* accepts V2 and trusts AD as a server. Therefore, AD can obtain the client’s personal information.

As explained above, using the stolen user’s password verifier, the adversary can impersonate the legal server and can easily find the user’s secret password PW using a password dictionary attack. Therefore, the SRP-6a protocol is insecure against stolen-verifier attacks.

### 4.3. Denning–Sacco Attack

The Denning–Sacco attack [41] occurs when an intruder obtains the session key from an eavesdropped session and uses it either to gain the ability to impersonate the user directly or to conduct a brute-force search against a long-term private key, such as the user’s password. This attack arises from the fact that compromising a fresh session key using an old key enables the protocol to be compromised.

In the SRP protocol, if the attacker can leak the session key $K_{C}$ from the client C, the following Denning–Sacco attack is possible:

1. Assume the attacker intercepts the request message (A||ID) sent by *C* to *S.*
2. To capture salt, the attacker starts an authentication session with the actual server S by forwarding parameters A and ID to S. S computes B and sends it to the attacker with the *salt* of client *C*.
3. The attacker can masquerade as a fake server, randomly generate a number b, guess any password $PW^{*}$, and compute his own parameters $x^{*}=H\left(salt,\;ID,PW^{*}\right)$ and $B^{*}=k\cdot g^{x^{*}}+g^{b}$. Then, they send $B^{*}$ and salt to C.
4. *C* computes the message $V1=H\left(p,\;g,ID,\;slat,\;A,\;B^{*},\;K_{C}\right)$ and sends it to the attacker, where $K_{C}=H\left({\left(B^{*}-K\cdot g^{x}\right)}^{a+u^{*}\cdot x}\right)$ and $u^{*}=H(A,B^{*})$.
5. Assuming that the attacker somehow obtained the shared session key $K_{SC}$ from the client, password PW can be obtained by performing the following offline password guessing attack:
  (i) The attacker makes a guess for the secret password $PW^{*}$ from a password dictionary.
  (ii) The attacker computes $x^{*}=H\left(salt,ID,PW^{*}\right)$, $u^{*}=H\left(A,\;B^{*}\right)$ and $K_{S}^{*}=H\left({\left(A\cdot g^{x^{*}\cdot u^{*}}\right)}^{b}\right)$ and checks if $K_{SC}=K_{S}^{*}.$
  (iii) If it holds, the attacker has guessed the correct secret password $PW^{*}=PW$. Otherwise, the attacker repeatedly performs the verification process.

Finally, the attacker can obtain the user’s secret password, which can be used to impersonate the client or the server.

## 5. Secure SIP

To secure SIP, this work introduces a new scheme, called secure-SIP (S-SIP). S-SIP consists of two protocols: the SIP authentication protocol (A-SIP) and the key management and protection protocol (KP-SIP). S-SIP provides SIP with essential security services to protect SIP messages against many attacks, which are authentication, integrity, confidentiality, and key management. The authentication service is provided to SIP using the A-SIP protocol, whereas the integrity, confidentiality, and key management services are provided using the KP-SIP protocol. The A-SIP protocol is an authentication protocol that provides mutual authentication for all entities in the VoIP system. KP-SIP is used to exchange session keys and authentication tickets between entities and to secure SIP signal messages exchanged between entities. The following explains these two protocols in detail.

### 5.1. A-SIP Protocol

**Figure 3:**
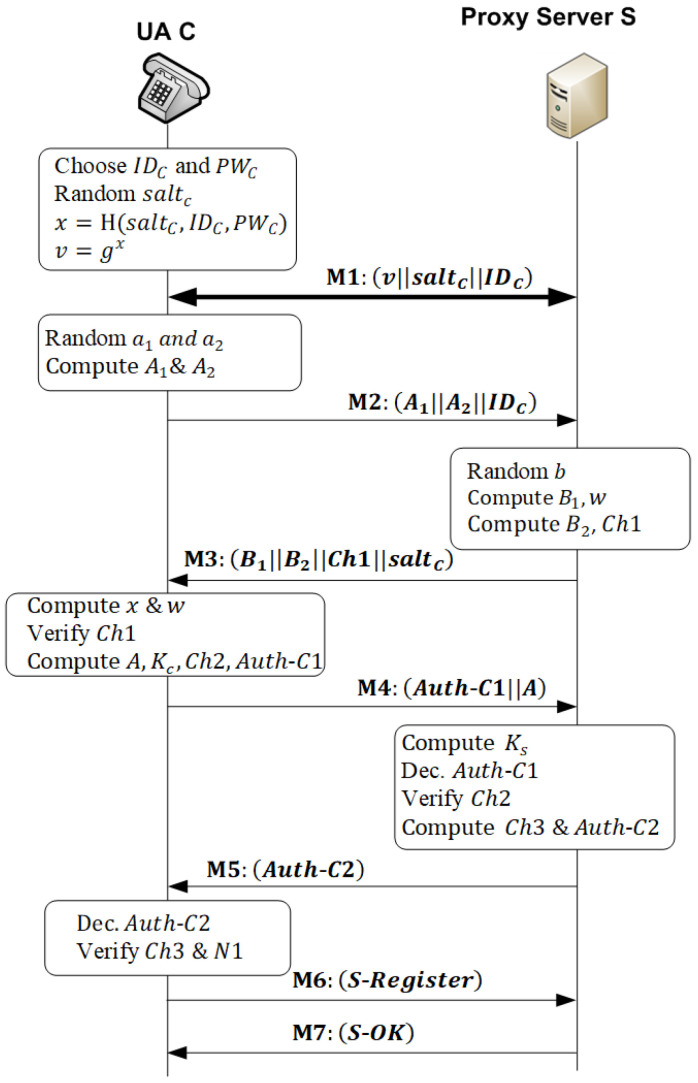
A-SIP protocol.

The A-SIP protocol is a novel mutual authentication scheme for the session initiation protocol. It is a client/server protocol based on the SRP protocol. However, it overcomes the flaws in the SRP protocol explained in Section 4. The A-SIP protocol is responsible for authenticating entities before the actual communication takes place between them. Figure 3 shows the proposed authentication protocol A-SIP. The used notations are listed in Table 1. The A-SIP protocol uses a four-way challenge/response handshake technique to provide mutual authentication. It consists of four phases: the setup phase, the login phase, the authentication phase and the registration phase. This section explains these phases in detail.

(1) Setup Phase

In this phase, the user is registered to the remote server S as a legal user by executing the following steps over a secure channel.

1. The user of a client *C* freely chooses their identity $ID_{C}$ and password $PW_{C}$. Then, *C* selects a random password $salt_{C}$ and then computes a verifier v and a secret exponent x as:
  $$\begin{array}{c} x=H\left(salt_{C}\left|\left|ID_{C}\right|\right|PW_{C}\right)\\ v=g^{x} \end{array}$$
2. *C* establishes a secure connection with S and sends v, $ID_{C}$, and $salt_{C}$ to S.
3. Upon receiving the message $M1=\left(v\left|\left|salt_{C}\right|\right|ID_{C}\right)$, S computes the password verifier $PW_{V}$ as $PW_{V}=v⊕H\left(ID_{C}||S_{P}\right)$, where $S_{P}$ is the private secret for the server, which is a random bit-string with high entropy. Finally, S stores $PW_{V}$ and $salt_{C}$ in its database indexed by $ID_{C}$.

(2) Login Phase

In the login phase, the user tries to login into the proxy server S to access different services over an insecure channel. As shown in Figure 3, the steps in this phase (messages M2 and M3) are executed as follows:

1. *C* generates two random numbers $a_{1}$ and $a_{2}$ from the range [1, q). Additionally, it computes two ephemeral public keys $A_{1}=g^{a_{1}}$ and $A_{2}=g^{a_{2}}$.
2. *C* sends the request message $M2=\left(A_{1}\left|\left|A_{2}\right|\right|ID_{C}\right)$ to the proxy server.
3. When *S* receives M2, it uses $ID_{C}$ to retrieve $salt_{C}$ and $PW_{V}$. Using $S_{P}$, *S* computes $H\left(ID_{C}||S_{P}\right)$, which is XORed with $PW_{V}$ to obtain v. Then, it generates a random number b∈[1, q) and computes the ephemeral public key $B_{1}=g^{b}$ and private key $w=H\left(v^{b}\left|\left|A_{1}\right|\right|A_{2}||salt_{C}\right)$. If *S* does not have credentials of *C*, it securely communicates with the authentication/registrar server to get them.
4. *S* computes the second ephemeral public key $B_{2}$ and the first challenge Ch1 as:
  $$B_{2}={\left(A_{1}\cdot A_{2}\cdot v\right)}^{b\cdot w}$$
  (1) $$Ch1=H\left(w\left|\left|B_{1}\right|\right|B_{2}\right)$$
5. *S* sends the message $M3=\left(B_{1}||B_{2}\left|\left|Ch1\right|\right|salt_{C}\right)$ to C.

(3) Authentication Phase

In the authentication phase, based on pre-shared parameters in the preceding phases, *S* and *C* verify the identity of each other. If they succeed, they generate and share a unique session key. Figure 3 shows the authentication phase between *C* and S (messages M4 and M5). The authentication procedure is performed on a common channel in the following steps:

1. Upon receiving the message M3, *C* computes x and w as:
  $$\begin{array}{c} x=H\left(salt_{C}\left|\left|ID_{C}\right|\right|PW_{C}\right)\\ w=H\left(B_{1}^{x}\left|\left|A_{1}\right|\right|A_{2}||salt_{C}\right)=H\left(g^{x\cdot b}\left|\left|A_{1}\right|\right|A_{2}||salt_{C}\right)=H\left(v^{b}\left|\left|A_{1}\right|\right|A_{2}||salt_{C}\right) \end{array}$$

Then, it computes the first challenge Ch1 using Equation (1).

2. *C* checks that the received challenge Ch1 is equal to the computed challenge. If not true, *C* terminates the session with S and does not complete the authentication process. Otherwise, *C* starts to compute the second challenge.
3. *C* computes the public key *A*, the session key $K_{C}$, and the second challenge Ch2 as:
  $$\begin{array}{c} A={\left(A_{1}\cdot B_{1}\cdot v\right)}^{a_{2}\cdot w}\\ K_{C}={\left(\frac{B_{2}}{B_{1}^{a_{2}\cdot w}}\right)}^{a_{2}}={\left(\frac{{\left(A_{1}\cdot A_{2}\cdot v\right)}^{b\cdot w}}{B_{1}^{a_{2}\cdot w}}\right)}^{a_{2}}={\left(\frac{{\left(A_{1}\cdot g^{a_{2}}\cdot v\right)}^{b\cdot w}}{g^{b\cdot a_{2}\cdot w}}\right)}^{a_{2}}= \end{array}$$
  (2) $$\;={\left(A_{1}\cdot v\right)}^{a_{2}\cdot b\cdot w}=g^{\left(a_{1}+x\right)a_{2}\cdot b\cdot w}$$
  $$Ch2=H\left(H\left(p\left|\left|g\right|\right|salt_{C}\right)⊕H\left(ID_{C}||B_{1}\right)⊕H\left(A\left|\left|B_{2}\right|\right|w\right)\right)$$
4. *C* generates a nonce N1, which is a random number that is difficult for an opponent to guess and represents a unique identifier for this transaction. *C* encrypts N1 and Ch2 using $K_{C}$ to construct the first authenticator $Auth\text{-}C1=E\left(K_{C},\;\left[Ch2||N1\right]\right)$ that represents the client authenticator. The subsequent message (response) received from *S* must contain the hash of the nonce N1.
5. *C* sends the challenge message M4=(Auth-C1||A) to *S*
6. When *S* receives the message, it computes its session key $K_{S}$ as:
  $$K_{S}={\left(\frac{A}{A_{2}^{b\cdot w}}\right)}^{b}={\left(\frac{{\left(A_{1}\cdot B_{1}\cdot v\right)}^{a_{2}\cdot w}}{A_{2}^{b\cdot w}}\right)}^{b}={\left(\frac{{\left(A_{1}\cdot g^{b}\cdot v\right)}^{a_{2}\cdot w}}{g^{a_{2}\cdot b\cdot w}}\right)}^{b}$$
  (3) $$={\left(A_{1}\cdot v\right)}^{a_{2}\cdot b\cdot w}=g^{\left(a_{1}+x\right)a_{2}\cdot b\cdot w}$$

From Equations (2) and (3), it is clear that $K_{S}=K_{C}=K_{SC}$, where $K_{SC}$ is the shared session key between *S* and *C*. Next, *S* decrypts Auth-C1 using *K_S_* to extract Ch2 and N1. Then, it computes the second challenge $Ch2^{*}$.

7. *S* compares the second challenge Ch2 extracted from Auth-C1 and the computed challenge $Ch2^{*}$. If $Ch2\neq Ch2^{*}$, *S* aborts the session with *C*. Otherwise, *S* computes the third challenge $Ch3=H\left(H\left(A||B_{2}\right)⊕H\left(Ch2||salt_{C}\right)\right)$
8. *S* encrypts Ch3 and H(N1) using session $K_{S}$ to construct the server authenticator as:
  $$Auth\text{-}C2=E\left(K_{S},\;\left[Ch3||H\left(N1\right)\right]\right)$$
9. After receiving the message M5=Auth-C2, *C* decrypts the message using $K_{C}$ to extract Ch3. Next, it verifies Ch3 and H(N1). If not correct, *C* terminates the sessions. Otherwise, *C* assures that the message has been sent by *S* and the authentication is successful. Using the nonce N1 assures *C* that this is a response for a fresh message and helps prevent a replay attack.

(4) Registration Phase

This is the last phase in the A-SIP protocol. In this phase, the client registers its URI address with the Proxy/Registrar SIP server at which the user can be reached. As explained in Section 3, registration with a local server is essential to receive or make an SIP call. The steps of this phase (messages M6 and M7) are as follows:

1. At the end of the authentication phase, *C* forms the *S-Register* message as
  $$S\text{-}\mathrm{Register}=E\left(K_{SC},\;\left[Register||H^{2}\left(N1\right)\right]\right)$$
  where Register is the standard SIP registration message and $H^{2}\left(N1\right)=H\left(H\left(N1\right)\right)$.
2. On receipt of the *S-Register* message, *S* decrypts the message to obtain the *Register* message and checks $H^{2}\left(N1\right)$. The SIP server processes the *Register* message to register the URI of the client *C* ($URI_{c}$).
3. *S* sends the *S-OK* message to *C*, which is computed as:
  $$\begin{array}{c} S\text{-}\mathrm{OK}=E\left(K_{SC},\;\left[OK\left|\left|H^{3}\left(N1\right)\right|\right|TKT_{CS}\right]\right)\\ TKT_{CS}=E\left(K_{P},\;\left[URI_{C}\left|\left|IP_{S}\right|\right|TS_{1}||LT_{1}\right]\right) \end{array}$$
  where OK is the standard SIP *OK* message, $H^{3}\left(N1\right)=H\left(H^{2}\left(N1\right)\right)$, and $TKT_{CS}$ is a ticket that *C* uses for subsequent authentications to obtain VoIP services from *S*. This is explained in detail in the next section.
4. When receiving the *S-OK* message from *S*, *C* decrypts it using the session key and checks $H^{3}(N1)$. If correct, *C* accepts the authentication ticket $TKT_{CS}$.

The ticket $TKT_{CS}$ contains the registered address of the client $URI_{C}$ and the network address of the proxy server $IP_{S}$. To prevent the adversary from reusing the ticket, it includes a timestamp $TS_{1}$, indicating the date and time at which the ticket was issued, and a lifetime $LT_{1}$, indicating the length of time for which the ticket is valid. In addition, the ticket is encrypted using the private key of the server $K_{P}$. Thus, it cannot be modified by *C* or by an opponent. The ticket helps minimize the number of times that a user has to enter a password. The ticket is reused for a single login session. For the lifetime of the ticket, *C* can use the ticket for multiple accesses to the same proxy server, as explained in the next section. If *C* moved to another region managed by another SIP server, it must register to the new SIP server to obtain a new authentication ticket.

### 5.2. KP-SIP Protocol

**Figure 4:**
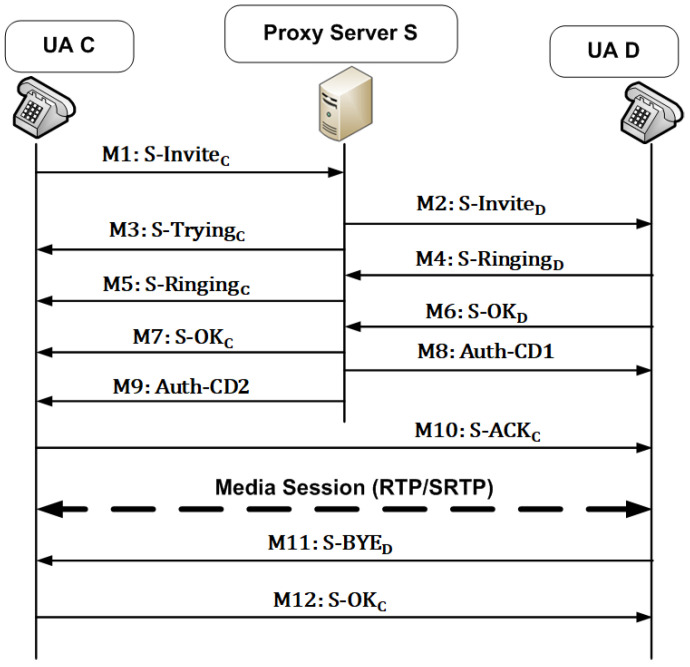
KP-SIP protocol.

The KP-SIP protocol is used for key management between entities, and for protecting SIP messages exchanged between different entities. Figure 4 explains the session example using KP-SIP when a single proxy is involved. Table 2 and Table 3 show the details of the exchanged messages shown in Figure 4. The KP-SIP protocol is divided into two phases: the call initiation phase (messages M1 to M10) and the call teardown phase (messages M11 and M12). Next, these phases are explained.

(1) Call Initiation Phase

Let user agent *C* attempt to call user agent *D*, where *C* and *D* are registered with proxy server *S*. Figure 4 shows the call flow. *C* initiates the session by sending the ${S\text{-}\mathrm{INVITE}}_{C}$ message to the proxy server. As shown in Table 2, ${S\text{-}\mathrm{INVITE}}_{C}$ contains the standard SIP invite message ${\mathrm{INVITE}}_{C}$ and the ticket obtained from the proxy server in the registration phase, the authenticator Auth-C3, the timestamp and the message lifetime. The message ${S\text{-}\mathrm{INVITE}}_{C}$ is encrypted using the last shared session key $K_{SC}$ with *S*. The authenticator Auth-C3 includes the URI of *C*, the network address $IP_{s}$ of *S*, and the nonce N2.

After receiving the ${S\text{-}\mathrm{INVITE}}_{C}$ message, *S* decrypts it using the last shared session key with *C*. Then, it checks $TS_{2}$ and $LT_{2}$ to ensure that the lifetime of the message has not expired. Then, it decrypts the ticket using its private key and verifies its validity using the timestamp and the lifetime included in the ticket. In addition, it compares $URI_{C}$ and $IP_{S}$ included in authenticator Auth-C3 with those included in the ticket. If the ticket is valid, $IP_{S}$ is correct, and $URI_{C}$ is correct and matches the registered URI of *C*, *S* authenticates *C* and processes the SIP INVITE message. To prevent a replay attack, the ${S\text{-}\mathrm{INVITE}}_{C}$ message has a very short lifetime compared to the ticket lifetime. Additionally, to prevent an opponent from replaying ticket $TKT_{CS}$, it is encrypted with the ${S\text{-}\mathrm{INVITE}}_{C}$ message using the session key.

The server sends the ${S\text{-}\mathrm{INVITE}}_{D}$ message to *D*, encrypted by the session key $K_{SD}$ shared between *S* and *D*. The ${S\text{-}\mathrm{INVITE}}_{D}$ message contains its lifetime $LT_{3}$, timestamp $TS_{3}$, and nonce N3 to avoid a replay attack.

The server sends the ${S\text{-}\mathrm{TRYING}}_{C}$ message to *C* that contains the standard SIP TRYING message and H(N2), which are encrypted using the session key $K_{SC}$. *S* returns the hash of N2 received in M1 to *C* to show the freshness of the reply. Every time *C* sends ${S\text{-}\mathrm{INVITE}}_{C}$, it starts a timer. If *C* receives ${S\text{-}\mathrm{TRYING}}_{C}$ before the timer expires, the timer is stopped, and the sender sends the next message. If the timer expires or N2 is not correct, *C* terminates the session and initiates another session by sending the ${S\text{-}\mathrm{INVITE}}_{C}$ message.

As shown in Figure 4 and Table 2, messages M4 to M7 consist of the standard SIP RINGING or OK messages and the hash of the last received nonce $(H^{j}\left(N\right)=H^{j-1}\left(N)\right)$. These messages are encrypted using the session keys shared between each user agent and the proxy server to protect them from alteration or spoofing. Additionally, the nonce is used to assure that the messages are fresh and have not been replayed by an adversary. *D* sends the message ${S\text{-}\mathrm{OK}}_{D}$ if it accepts the call. *S* forwards the SIP OK message to *C* using the ${S\text{-}\mathrm{OK}}_{C}$ message.

For mutual authentication between *C* and *D*, the server sends the authenticators Auth-CD1 and Auth-CD2, as shown in Figure 4. For message integrity and mutual authentication, the contents of Auth-CD1 and Auth-CD2 are encrypted using the shared session key between each user agent and the server, as shown in Table 3. Auth-CD1 and Auth-CD2 contain a copy of the session key $K_{CD1}$, which is used for protecting messages exchanged directly between *C* and *D*. Additionally, they have several pieces of information: URI of *C*, the network address of the server $IP_{S}$, the hash of the last nonce exchanged with the server, the nonce *N*4, the timestamp, and the lifetime of the message. As explained above, these pieces are included to prevent spoofing and replaying attacks.

At the end of the call initiation phase, *C* sends the ${S\text{-}\mathrm{ACK}}_{C}$ message to *D*. The contents of this message are encrypted using the shared session key $K_{CD1}$ sent by *S* in the authenticators Auth-CD1 and Auth-CD2 in messages M8 and M9. The ${S\text{-}\mathrm{ACK}}_{C}$ message contains the standard SIP ACK message, the hash of N4 proposed by *S* in the authenticators. Additionally, it contains a new random session key $K_{CD2}$ suggested by *C*. Encrypting the content of the ${S\text{-}\mathrm{ACK}}_{C}$ message using $K_{CD1}$ and exchanging N4 assures mutual authentication and prevents a replay attack. After receiving ${\;S\text{-}\mathrm{ACK}}_{C}$, *D* can extract and process the standard SIP ACK message to start a media session with *C*. Protecting media streams is out of the scope of the proposed work.

(2) Teardown Phase

In this phase, one of the user agents ends the SIP session by sending the S-BYE message. The other user agent responds with the S-OK message. These messages include the standard SIP BYE and OK messages. Additionally, to prevent a replay attack, the S-BYE message includes the hash of the nonce received in the messages M10, the timestamp, and the lifetime. The contents of these messages are encrypted using the last session key $K_{CD2}$ shared between *C* and *D*.

**Figure 5:**
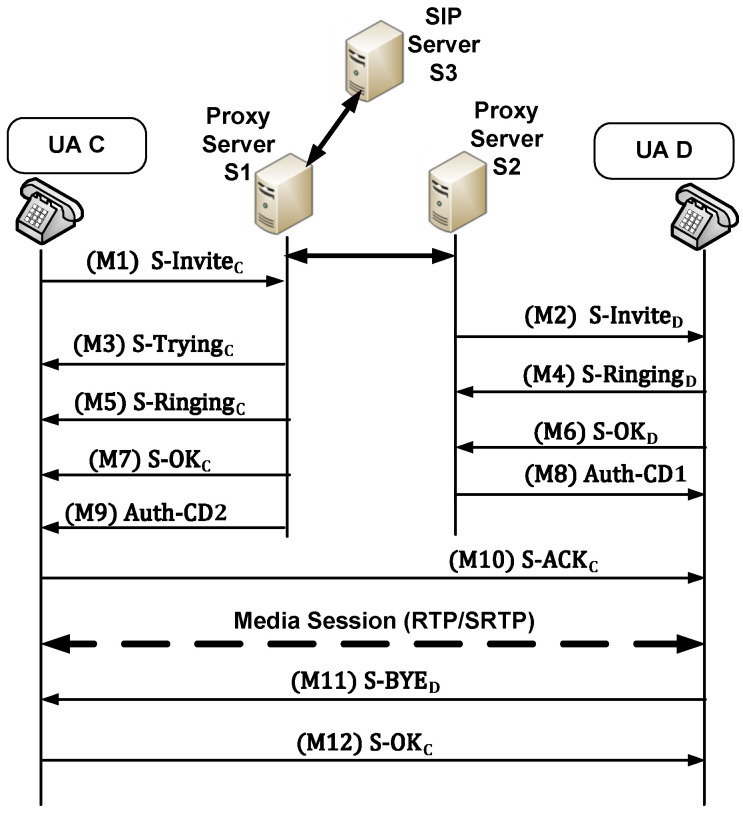
KP-SIP protocol with two proxy servers.

If the record-route option is enabled, the BYE and OK messages are routed through proxy servers, as explained in Section 3. In this case, the BYE and OK messages are encrypted using the session key exchanged between the user agents and the server ($K_{SC}$ and $K_{SD}$). If the messages exchanged between UA1 and UA2 are routed through multiple SIP servers, as shown in Figure 5, we suppose that the connections between SIP servers are protected using symmetric or asymmetric cryptography algorithms. In addition, routing messages through servers owned by different telecoms/providers is out of scope this work. KP-SIP can be extended to protect connections between different SIP servers.

## 6. Informal Security Analysis

In this section, we prove that the proposed protocol S-SIP is secure and can withstand various attacks and provide security requirements for SIP as follows. Similar to related studies, we assume that the adversary/attacker AD can insert, capture, delete, or modify any messages in the public insecure channel.

### 6.1. Offline Password Guessing Attack

As explained in Section 4, to perform an offline password guessing attack, an active adversary AD eavesdrops the communication flows between *C* and *S* and masquerades as a fake server or client. AD tries to modify and intercept any message that can be exploited to perform the attack. Then, the adversary goes offline and uses a dictionary to test passwords against the intercepted messages. If AD tries to impersonate the server, the attack is performed as follows:

*1*.  During the authentication process, AD intercepts the request message $M2=\left(A_{1}\left|\left|A_{2}\right|\right|ID_{C}\right)$ sent by *C* to login to S.
*2*.  The attacker forwards M2 to *S* to obtain $salt_{C}$. Then, they generate a random number b, guesses any password $PW^{*}$, and computes their own parameter as:
  $$\begin{array}{c} x^{*}=H\left(salt_{C}^{*}\left|\left|ID_{C}\right|\right|PW^{*}\right)\\ w^{*}=H\left(g^{x^{*}\cdot b}\left|\left|A_{1}\right|\right|A_{2}||salt_{C}\right)\;\\ B_{2}^{*}={\left(A_{1}\cdot A_{2}\cdot g^{x^{*}}\right)}^{b\cdot w^{*}}\\ Ch1^{*}=H\left(w^{*}\left|\left|B_{1}\right|\right|B_{2}^{*}\right) \end{array}$$

Then, they send $M3=(B_{1}||B_{2}^{*}\left|\left|Ch1^{*}\right|\right|salt_{C})$ to C

*3*.  Upon receiving M3, *C* computes x, w, and Ch1. Next it compares the received challenge $Ch1^{*}$ and the computed challenge Ch1. If the guessed passwords $PW^{*}$ are not correct, *C* detects that $Ch1^{*}\neq Ch1$. Therefore, *C* terminates the connection with the fake server.

As shown in the former steps, AD did not receive any message that can be used to check the correctness of the guessed parameters. Therefore, AD cannot perform an offline password guessing attack.

If AD tries to impersonate the client with user identity $ID_{C}$, the attack is performed as follows:

1. *AD* randomly generates $a_{1}$ and $a_{2}$ and calculates public keys $A_{1}$ and $A_{2}$ and then transmits them to S with user identity $ID_{C}$.
2. S randomly generates a random number b, calculates public keys $B_{1}$ and $B_{2}$, the secret parameter w, and the challenge Ch1. Next, it sends $B_{1}$, $B_{2}$, $salt_{C}$, and Ch1 to AD.
3. AD guesses any password $PW^{*}$ and computes other parameters:
  $$\begin{array}{c} x^{*}=H\left(salt_{C}\left|\left|ID_{C}\right|\right|PW^{*}\right)\\ w^{*}=H\left(B_{1}^{x^{*}}\left|\left|A_{1}\right|\right|A_{2}||salt_{C}\right)\\ A^{*}={\left(A_{1}\cdot B_{1}\cdot g^{x^{*}}\right)}^{a_{2}\cdot w^{*}}\\ K_{c}^{*}={\left(B_{2}/B_{1}^{a_{2}\cdot w^{*}}\right)}^{a_{2}}\\ Ch2^{*}=H\left(H\left(p\left|\left|g\right|\right|salt_{c}\right)⊕H\left(ID_{C}||B_{1}\right)⊕H\left(A^{*}\left|\left|B_{2}\right|\right|w^{*}\right)\right)\\ Auth\text{-}C1^{*}=E\left(K_{c}^{*},\;\left[Ch2||N1\right]\right) \end{array}$$

Then, AD sends the message $M4=(Auth\text{-}C1^{*}||A^{*})$ to *S*.

4. Upon receiving M4, S encrypts the message using its key $K_{s}={\left(A^{*}/A_{2}^{b\cdot w}\right)}^{b}$. If the guessed password and secret key are wrong, *S* detects that $Auth\text{-}C1^{*}\neq \;Auth\text{-}C1$. Therefore, S does not respond to *AD* and terminates the session.

As clear in this attack, AD did not obtain any response message from the server that can be used to perform an offline password guessing attack. As a result, impersonation as a legal client or server does not enable the attacker to perform an offline password guessing attack. Therefore, the proposed authentication protocol is not vulnerable to offline password guessing attacks.

### 6.2. Denning–Sacco Attack

As explained in Section 4, a Denning–Sacco attack refers to obtaining a long-term key such as the user’s password or the session key through an obtained old session key. The attacker tries to guess either the user’s password or the session key using an old, compromised session key. In the proposed protocol, as shown in Equations (2) and (3), the session key is computed as

$$K_{C}=K_{S}={\left(B_{2}/B_{1}^{a_{2}\cdot w}\right)}^{a_{2}}={\left(A/A_{2}^{b\cdot w}\right)}^{b}$$

where $w=H\left(B_{1}^{x}\left|\left|g^{a_{1}}\right|\right|g^{a_{2}}||salt_{C}\right)$. The random numbers $a_{1}$, $a_{2}$, and b are changed for every session. Therefore, if a passive eavesdropper acquires an old session key, they are not able to compute the server’s or client’s session key. In addition, the attacker cannot perform offline password guessing attacks using the old session key due to the difficulty of the computational Diffie–Hellman problem, which is computationally infeasible with large random numbers.

Let the active eavesdropper AD acquire the session key $K_{SC}$ from the client. Let the eavesdropper impersonate server *S* and perform a Denning–Sacco attack explained in Section 4.3 as follows:

1. During the authentication process, AD intercepts the request message $M2=\left(A_{1}\left|\left|A_{2}\right|\right|ID_{C}\right)$ sent from *C* to S. Then, they forward M2 to *S* to obtain $salt_{C}$.
2. AD generates a random number b, guesses any password $PW^{*}$, and computes their own parameter as:
  $$\begin{array}{c} x^{*}=H\left(salt_{C},\;ID_{C},PW^{*}\right)\\ w^{*}=H\left(g^{x^{*}\cdot b}\left|\left|A_{1}\right|\right|A_{2}||salt_{C}\right)\;\\ B_{2}^{*}={\left(A_{1}\cdot A_{2}\cdot g^{x^{*}}\right)}^{b\cdot w^{*}}\\ Ch1^{*}=H\left(w^{*}\left|\left|B_{1}\right|\right|B_{2}^{*}\right) \end{array}$$

Then, they send $M3=\left(B_{1}^{*}\left|\left|B_{2}^{*}\right|\right|Ch1^{*}\right)$ to C.

3. To perform the brute-force attack using the compromised session key $K_{SC}$, AD must first compute the server session key as $K_{S}^{*}={\left(A/A_{2}^{b\cdot w^{*}}\right)}^{b}$. To compute $K_{S}^{*}$, AD must obtain A from *C*.
4. Upon receiving M3, *C* computes x, w, and *Ch*1. Next, it compares the received and computed challenges (Ch1 and $Ch1^{*})$. If the guessed password $PW^{*}$ is not correct, *C* detects that $Ch1^{*}\neq Ch1$. Therefore, *C* terminates the connection with the server and does not send the parameter A to AD.

In step 4, because the client closed the connection with AD, they could not obtain $A={\left(A_{1}\cdot B_{1}\cdot v\right)}^{a_{2}\cdot w}$ from C. AD cannot compute A or $a_{2}$ using the public key $A_{2}$ because they have will face the difficulty of the discrete logarithm problem. Thus, the proposed protocol is not vulnerable to Denning–Sacco attacks.

### 6.3. Stolen-Verifier Attack

As explained in Section 4, a stolen-verifier attack occurs when an adversary who steals the password-verifier from the server impersonates a legitimate user in the authentication process. Additionally, he may mount a guessing attack to retrieve the user’s password. 

For the proposed protocol, the server stores ID and $PW_{V}=v⊕H\left(ID_{C}||S_{P}\right)$ for each client in its database. Let an impersonator be the adversary who steals the password verifier $PW_{V}$ and $ID_{C}$ of *C* from the database. Then, they try to perform an offline password guessing attack. The adversary chooses a secret password $PW^{*}$ from a password dictionary. Then, they compute $x^{*}=H\left(salt_{C},ID_{C},PW^{*}\right)$ and $v^{*}=g^{x^{*}}$. Next, they try to guess the private key of the server as $S_{P}^{*}$ to compute $PW_{V}^{*}=g^{x^{*}}\;⊕H\left(ID_{C}||S_{P}^{*}\;\right)$. The process is repeated if $PW_{V}^{*}\neq PW_{V}$. This attack is not feasible because $S_{P}$ has high entropy and cannot be guessed. Therefore, AD cannot retrieve v or x from $PW_{V}$. Thus, they cannot compute the correct values for $w^{*}=H\left(g^{x^{*}\cdot b}\left|\left|A_{1}\right|\right|A_{2}||salt_{C}\right)$, $B_{2}^{*}={\left(A_{1}\cdot A_{1}\cdot v^{*}\right)}^{b\cdot w^{*}}$ and $Ch1^{*}=H\left(w^{*}\left|\left|B_{1}\right|\right|B_{2}^{*}\right)$. If AD sent an incorrect value of $Ch1^{*}$, *C* closes the connection with AD. Therefore, the adversary cannot impersonate the server even if they have the password verifier stored in the server. In addition, they cannot obtain any information from the client to be exploited to perform an offline password guessing attack.

### 6.4. Perfect Forward Secrecy

Perfect forward secrecy means that if session keys of one or more entities are compromised, the secrecy of old session keys established by the trusted entities are not affected. It also means that a stolen session key does not help an attacker perform a password guessing attack.

In the proposed protocol, as explained in Section 5, the session key $K_{SC}$ is computed using random numbers (private keys) $a_{1}$, $a_{2}$, and b. These random numbers are changed every session. Therefore, if $K_{SC}$ is compromised, adversary AD cannot obtain the session keys of past sessions. In addition, as explained in Section 6.2, if the session key is compromised, the AD cannot perform offline password guessing attacks. Thus, the proposed protocol has the properties of perfect forward secrecy.

### 6.5. Impersonation Attack

In impersonation (spoofing) attacks, the adversary tries to masquerade as a legitimate user or server. As explained in Section 6.1 in detail, for the proposed protocol, the adversary cannot perform an offline password guessing attack because they are unable to impersonate either the user or the server. The client or the server closes the connection with the other party if one of the challenges, Ch1 or Ch2, or authenticators, Auth-C1 or Auth-C2, are not valid. To impersonate the user, AD must obtain access to the user’s password. However, to impersonate the server, AD must obtain access to the server’s secret $S_{P}$. However, these values are kept secret. Consequently, the attacker cannot impersonate the user or the proxy server.

### 6.6. Replay Attack

A replay attack can be performed if the adversary replays any eavesdropped or intercepted message to forge any legitimate participant. In the A-SIP protocol, the adversary can replay the login request (M2) or replay (M3) to impersonate the user or the server. However, as explained above, the adversary cannot generate authenticators, Auth-C1 or Auth-C2, or the session key, $K_{SC}$, as shown in Equations (2) and (3). This is because it is not feasible to recover $a_{1}$, $a_{2}$, b, x, or v from $A_{1}$, $A_{2}$, A, $B_{1}$, $B_{2}$, and Ch1. Consequently, the adversary fails to authenticate themselves to the client or the server by replaying the login request. Therefore, a replay attack is not applicable for the A-SIP protocol. For the KP-SIP protocol, this type of attack is not possible. This is because the identity of users is protected by encrypting all messages using different session keys.

### 6.7. Session Teardown Attack

If the attacker discovers credential information from the INVITE message, they can prepare a false BYE message to be sent to the proxy server or one of the user agents to terminate the VoIP session. However, using the S-SIP protocol prevents this attack. User agent *C* or *D* uses the session key ($K_{CD2}$) generated during the authentication process to encrypt the BYE message sent by the caller or callee user agents. Therefore, another party (*C* or *D*) can verify the received message. The user agent decrypts the message using the secured shared key $K_{CD2}$. Then, checks N4. If correct, the client processes the BYE message and terminates the session. 

### 6.8. Registration Hijacking Attack

To perform this attack, the adversary must impersonate a valid user agent such as *C*. The adversary sends an SIP registration message to the proxy server including the URI of *C*. However, to do so, the adversary must be authenticated at the proxy server through the authentication phase. Then, they encrypt the SIP registration message using the session key and send it to the server. However, as explained in detail in Section 6.1, impersonation of a valid user is not possible. Therefore, the adversary cannot authenticate themselves to the proxy server and cannot obtain the session key. Thus, S-SIP is not vulnerable to registration hijacking attacks.

### 6.9. Request Spoofing Attack

In this attack, the attacker sends a spoofed INVITE message to fool a legitimate recipient who may believe that they are communicating with another known entity. With the proposed solution, spoofing the S-INVITE message is not possible. Before transmitting the INVITE message, any client must start the process of the mutual authentication phase. If the authentication process fails, the client cannot obtain an authentication ticket or session key. Therefore, because the client’s ticket and identity are encrypted using the shared session key, the client cannot construct the S-INVITE message and cannot initiate an SIP call using different identities. Thus, the proposed solution can resist a request spoofing attack.

### 6.10. Message Tampering Attack

Message tampering attacks occur when an attacker intercepts and modifies packets exchanged between SIP components. This attack is not available in the proposed solution. As shown in Table 2 and Table 3, the most sensitive information that the attacker needs to perform this attack is protected by encryption using shared session keys between entities. 

### 6.11. Man-In-The-Middle Attack

The MITM attack is one of the most serious threats to the security and trust of existing VoIP protocols and systems. Using this attack, the attacker can easily wiretap, divert and even hijack VoIP calls by tampering with VoIP signaling and/or media traffic. To execute the MITM attack, the attacker masquerades as a client and an SIP server. However, as explained in Section 6.5, the proposed solution prevents client or server impersonation attacks. Thus, the proposed protocol is not vulnerable to MITM attacks.

### 6.12. Re-INVITE Attack

After a session is established between user agents, one of them can send a SIP Re-INVITE message to modify the parameters of the session. Therefore, the attacker can perform a DoS attack using the Re-INVITE messages by sending it to one of the user agents. However, as explained above, the S-SIP protocol protects all SIP messages exchanged between user agents and prevents user impersonation attacks. Thus, it prevents Re-INVITE attacks.

## 7. Formal Security Analysis

ProVerif [53] is a tool used for automatic verification of cryptographic protocols, which is widely used to analyze the security of authentication and key agreement protocols [49,65,66,67,68]. In this section, a simulation of the proposed protocols described in Section 5 is performed using ProVerif to illustrate its robustness and correctness under a formal and automated threat model.

Based on the applied π calculus, ProVerif is used to verify key security requirements such as authentication, secrecy, anonymity, and privacy. It can support modeling many cryptographic primitives including one-way functions, encryption and decryption (symmetric and asymmetric), digital signatures, Diffie–Hellman key agreements and many more. Moreover, ProVerif can simulate various attacks. A-SIP and KP-SIP protocols were verified using ProVerif.

For the A-SIP protocol, the complete ProVerif model can be found in [69], and the following explains the main parts of the model in detail, which are shown in Appendix A. We initially defined two channels: a secure channel *SCh* that was used for secure communication between the client and the server, and a public channel *Ch* that was used for public/insecure communication between the client and the server. *SCh* was used in the setup phase and *Ch* was used in the login, authentication, and registration phases. Then, shared session keys, constants, variables, and types used in the proposed A-SIP protocol were defined.

ProVerif defines cryptographic primitives as constructors, destructors and equations. So, the needed constructors (functions) were defined, such as exp, xor, concat, sEnc, and sDEC. Then, the required equations were defined to model the properties of exclusive-OR, modular exponentiation, symmetric encryption, symmetric decryption, extract the first concatenated values, and extract the second concatenated values. Next, four events (UserStarted, UserAuthed, ServerStarted, and ServerEnd) were defined to model the initiation and termination of both client and server processes. The events were used to analyze the security of the proposed protocol.

For A-SIP, we modeled all interactions between the client and the server using two processes: one for the client (ProcessClient) and one for the server (ProcessServer). The ProVerif script for A-SIP, which included these processes, can be found in [69]. To verify the model, the two participants could interact by establishing many sessions. Therefore, these two processes were replicated for unbounded parallel executions as follows:
$$\begin{array}{c} \left(*========\text{\textbackslash{}}\mathrm{Main}========*)\right\}\\ process\;\left(!ProcessServer|!ProcessClient\right) \end{array}$$

Finally, six queries were defined to verify the correctness of the proposed scheme and session key secrecy. These six queries were applied in the main part.
$$\begin{array}{c} \left(*=====Queries=====*\right)\\ query\;attacker\left(Kc\right).\\ query\;attacker\left(Ks\right).\\ query\;attacker\left(Ksc\right).\\ query\;attacker\left(Kp\right)\\ query\;id:\mathrm{bitstring};\;inj\text{-}event\left(\mathrm{UserAuthed}\left(id\right)\right)\;\Rightarrow inj\text{-}event\left(\mathrm{UserStarted}\left(id\right)\right).\\ query\;id:\mathrm{bitstring};\;inj\text{-}event\left(\mathrm{ServerEnd}\left(id\right)\right)\;\Rightarrow inj\text{-}event\left(\mathrm{ServerStarted}\left(id\right)\right). \end{array}$$

ProVerif performed an unbounded number of executions for the model to verify the authenticity and reachability. We executed the modeled processes in ProVerif 2.02. The verification results are as follows:

1. Weak secret PWc is true.
2. Query not attacker(Kc[]) is true.
3. Query not attacker(Ks[]) is true.
4. Query not attacker(Ksc[]) is true.
5. Query not attacker(Kp[]) is true.
6. Query inj-event(UserAuthed(id)) ==> inj-event(UserStarted(id)) is true.
7. Query inj-event(ServerEnd(id)) ==> inj-event(ServerStarted(id)) is true.

When we defined the password as a weak secret, ProVerif tried to prove that the attacker cannot distinguish a correct guess of the secret from an incorrect guess. Therefore, result number 1 shows that the proposed scheme can suppress a password guessing attack. The result numbers 2 to 5 verify that the session keys *K_C_*, *K_S_*, *K_SC_*, or *K_P_* were not revealed to the adversary and that secrecy was maintained. The result numbers 6 and 7 show that both ProcessClient and ProcessServer processes initiated and were completed successfully, respectively, which illustrates the correctness of the proposed authentication protocol.

For the KP-SIP protocol, the complete ProVerif model can be found in [69]. In the ProVerif script, seven queries were defined to verify the correctness of KP-SIP and the session key secrecy. The seven queries were applied in the main part and are as follows:
$$\begin{array}{c} \left(*========Queries========*\right)\\ query\;attacker\left(Ksc\right).\\ query\;attacker\left(Ksd\right).\\ query\;attacker\left(Kcd1\right).\\ query\;attacker\left(Kcd2\right).\\ query\;URIc:\mathrm{bitstring};\;inj\text{-}event\left(\mathrm{C\_End}\left(URIc\right)\right)\;\Rightarrow inj\text{-}event\left(\mathrm{C\_End}\left(URIc\right)\right).\\ query\;URId:\mathrm{bitstring};\;inj\text{-}event\left(\mathrm{C\_End}\left(URId\right)\right)\;\Rightarrow inj\text{-}event\left(\mathrm{C\_End}\left(URId\right)\right).\\ query\;id:\mathrm{bitstring};\;inj\text{-}event\left(\mathrm{ServerEnd}\left(id\right)\right)\;\Rightarrow inj\text{-}event\left(\mathrm{ServerStarted}\left(id\right)\right). \end{array}$$

After performing an unbounded number execution for the model to verify the authenticity and reachability, ProVerif showed the following verification results for the KP-SIP model:

1. Query not attacker(Ksc[]) is true.
2. Query not attacker(Ksd[]) is true.
3. Query not attacker(Kcd1[]) is true.
4. Query not attacker(Kcd2[]) is true.
5. Query inj-event(C_End(URIc_1)) ==> inj-event(C_End(URIc_1)) is true.
6. Query inj-event(C_End(URId_2)) ==> inj-event(C_End(URId_2)) is true.
7. Query inj-event(ServerEnd(id)) ==> inj-event(ServerStarted(id)) is true.

The result numbers 1 to 4 verify that the session keys *K_SC_*, *K_CD1_*, *K_SD_*, or *K_CD2_* were not revealed to the adversary and that secrecy was maintained. The result numbers 5 to 7 show that all processes for *C*, *D* and *S* initiated and were completed successfully, which illustrates the correctness of the proposed protocol.

## 8. Performance Analysis

### 8.1. Performance Comparison

In this section, we compare the security features and performance of the proposed scheme with other related schemes [39,41,42,43,45,48,49,50,51]. First, we compared the proposed authentication protocol A-SIP with related protocols considering various security features. Table 4 demonstrates the analysis of the security features for A-SIP in comparison with the related works. The proposed protocol was secure against all mentioned attacks, and can provide security requirements, including perfect forward secrecy and stolen-verifier attacks. In other words, the proposed scheme provides a high level of security compared to the related authentication protocols.

For performance comparison, the computational cost for each related authentication protocol was calculated using the primitive arithmetic and cryptographic operation timings. Table 5 shows the notation used for different cryptographic operations and the arithmetic mean and standard deviation of the computation cost of each operation. These cryptographic operations were implemented using Python programming language using the PyCryptodome 3.11.0 library on a personal computer with 8 GB RAM, an Intel Core i5-10210U CPU @ 2.1 GHz, and a 64-bit Windows 10 Professional operating system. Each operation was executed thousands of times until we obtained a 95% confidence interval with 2% error.

To compute the computational cost of symmetric encryption and decryption, we used AES with a 256-bit key and a message with a size of 512 bytes. For ECC point multiplication and addition, the standard elliptic curve Secp256k1 was used, where the modulus *p* and the order *n* were 256-bit numbers. This curve provides 128-bit security strength and is one of the curves adopted by many applications, such as OpenSSH and Bitcoin.

In authentication schemes, the login and authentication phases are executed more frequently than other phases that are performed only once. Therefore, the computational costs of only the login and authentication phases were considered in the performance comparison. A detailed performance evaluation of each related scheme is shown in Table 6. This table shows the arithmetic and cryptographic operations performed on both the client side and the server side and the estimated computational cost for each protocol. The timings in Table 6 were calculated using the primitive arithmetic and cryptographic operation timings given in Table 5. The computational cost of lightweight operations, such XOR and concatenation, were ignored.

As shown in Table 6, the proposed scheme was fourth in terms of computational cost. The authentication schemes introduced in [50,51] had the lowest costs. However, as explained in Section 2, these schemes do not provide the perfect forward secrecy security requirement.

### 8.2. S-SIP Overhead

**Figure 6:**
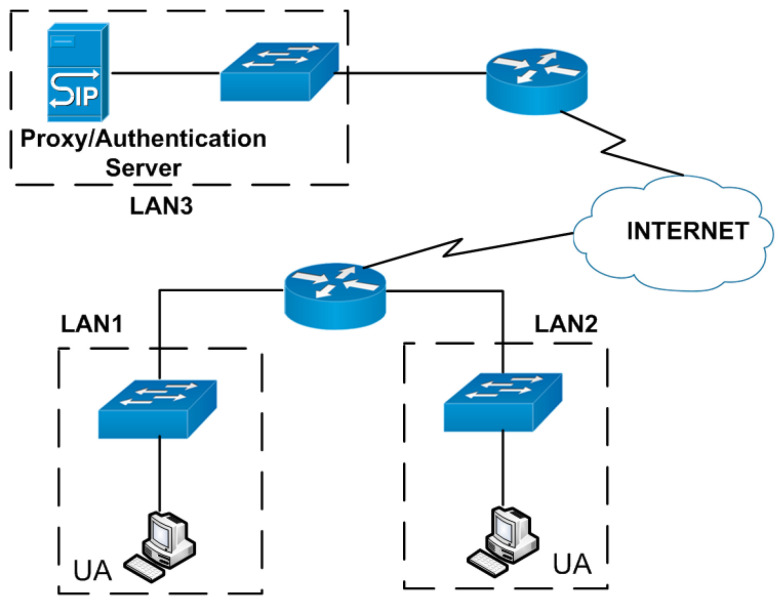
Testbed network architecture.

To characterize the performance of the S-SIP protocol and to indicate its overhead compared to SIP and related protocols based on TLS, we implemented an experimental testbed shown in Figure 6 based on the scenario depicted in Figure 1A. The testbed was a wide area network consisting of three 100 Mbps LANs. The user agents were connected to the first and second LANs, whereas the proxy/authentication server resided on the third LAN. As shown in Figure 6, the user agents and the server were connected through the internet over 500 Mbps connections.

The two user agents had the same specifications explained in Section 8.1. The server was a PC that had an Intel core i7 2.4 GHz processor and 16 GB RAM with a Windows 10 Pro 64-bit operating system. The S-SIP protocol was implemented using Python. The user agents and the server were implemented based on the scenario shown in Figure 1, where exchanging RTP messages were not considered because it was out of the scope of this work. In all experiments, for symmetric encryption and decryption, we used AES with a 256-bit key.

To compare between S-SIP and TLS-based techniques used to secure SIP, we suppose that SIPS/TLS is used for securing SIP messages and SRP is used as a password-based authentication protocol. We called this method as SRP–TLS. SRP–TLS was implemented using Python, where TLS version 1.2 with cipher suit ECDHE-RSA-AES256-GCM-SHA384 were used.

Because S-SIP and SRP–TLS use more messages than SIP, their overhead depends on the message round trip time (RTT) between clients and the server. Therefore, we considered two scenarios: small and large RTT. Thus, the server was placed in an area geographically close or far from the user agents to represent these two scenarios. 

The overheads of S-SIP and SRP-TLS were measured using two performance metrics: the authentication and registration time *T_A_* and the call setup and teardown time *T_S_*. *T_A_* is the time required to authenticate and register a user agent to the server. *T_S_* is the time required to setup and tear down the call between the caller and callee.

For all measured performance metrics, experiments were repeated until we obtained 95% confidence intervals with 2% error. For S-SIP, SRP–TLS and SIP, the mean and standard deviation were measured for each performance metric. To measure the actual overhead, we suppose that SIP does not use any authentication mechanism. For small and large RTT scenarios, the measured ping times between the user agents and the server were 32 and 227 ms, respectively. The results are shown in Table 7.

Compared to *T_S_*, the overhead in *T_A_* was larger mainly due to the authentication process and handshake mechanisms supported by S-SIP and SRP–TLS. For small RTT, the total overheads of S-SIP and SRP-TLS were 132.3 and 256.1 ms, respectively, whereas for large RTT, they were 621.1 and 1230.4 ms, respectively. In the case of small and large RTTs, the total overhead of SRP–TLS was nearly double that of S-SIP. The main overhead in the SIPS protocol was due to the TLS handshake phase and verification of digital certificates. In addition, SIPS protocol constructs TLS secure tunnels among each hop in the path from the client to the final recipient. Therefore, increasing the number of proxy servers between user agents greatly increased the overhead of SIPS.

## 9. Conclusions

This work introduced a new protocol for securing SIP called S-SIP, which can thwart most SIP attacks. S-SIP consists of two protocols: A-SIP and KP-SIP. The A-SIP protocol is an authentication protocol that provides mutual authentication for SIP entities. The KP-SIP protocol is used to secure SIP signaling messages and to exchange session keys and authentication tickets between SIP entities. A-SIP is based on the SRP protocol, which is one of the standard password-based authentication protocols supported by TLS. We informally analyzed the security issues of SRP. We showed that it is vulnerable to offline password guessing, stolen-verifier, and Denning–Sacco attacks. Therefore, we proposed the A-SIP protocol to overcome these security flaws in SRP. In addition, through informal security analysis, we showed that S-SIP can thwart many SIP-based attacks, such as session teardowns, registration hijacking, request spoofing, message tampering, and re-invite attacks. Additionally, we verified the security of S-SIP through formal analysis using the ProVerif tool. Moreover, we compared A-SIP with multiple related authentication schemes in terms of security and performance. Comparisons showed that A-SIP has more security features and lower computational costs. SIPS is a standard protocol for securing SIP based on TLS. To characterize the overhead of S-SIP compared to SIP and SRP–TLS, its performance was analyzed. Compared to SIP, in the case of small and large RTTs, the overheads of S-SIP were 132.3 and 621.1 ms, respectively. For SRP–TLS, for small and large RTTs, the overheads were 256.1 and 1230.4 ms, respectively. The results showed that the performance of S-SIP was better than SRP-TLS. In addition, increasing the number of proxy servers between user agents greatly increased the overhead of SRP–TLS. As a future work, KP-SIP can be extended to secure connections among SIP servers owned by the same or different telecoms/providers.

## Figures and Tables

**Table 1:** Notations.

Notation	Description
p	Large prime number
g	Primitive root modulo *p* (generator)
H(⋅)	One-way hash function
$PW_{C}$	Password of client C
$K_{Y}$	Session key of entity Y
$K_{XY}$	Session key shared between X and Y
a, b	High-entropy random numbers
||	Concatenation operation
$TS_{i}$	Timestamp number *i*
$LT_{i}$	Lifetime number *i*
$N_{i}$	Nonce number *i*
⊕	Bitwise XOR operation
↔	Secure channel
→	Common channel
*ID_C_*	*ID* of entity *C*
E(*K*, [*M*])	Symmetric encryption of *M* with the key *K*

**Table 2:** Messages M1 to M7 of the KP-SIP protocol.

(M1) $\;{S\text{-}\mathrm{INVITE}}_{C}=E\left(K_{SC},\;\left[{\mathrm{INVITE}}_{c}\left|\left|Auth\text{-}C3\right|\right|TKT_{CS}\left|\left|\;TS_{2}\right|\right|LT_{2}\right]\right)$ $Auth\text{-}C3=\left(URI_{C}\left|\left|IP_{S}\right|\right|N2\right)$ (M2) ${S\text{-}\mathrm{INVITE}}_{D}=E\left(K_{SD},\;\left[{\mathrm{INVITE}}_{D}\left|\left|N3\right|\right|TS_{3}||LT_{3}\right]\right)$ (M3) ${S\text{-}\mathrm{TRYING}}_{C}=E\left(K_{SC},\;\left[{\mathrm{TRYING}}_{C}||H\left(N2\right)\right]\right)$ (M4)${\;S\text{-}\mathrm{RINGING}}_{D}=E\left(K_{SD},\;\left[{\mathrm{RINGING}}_{D}||H\left(N3\right)\right]\right)$ (M5)${\;S\text{-}\mathrm{RINGING}}_{C}=E\left(K_{SC},\;\left[{\mathrm{RINGING}}_{C}||H^{2}\left(N2\right)\right]\right)$ (M6)${\;S\text{-}\mathrm{OK}}_{D}=E\left(K_{SD},\;\left[{\mathrm{OK}}_{D}||H^{2}\left(N3\right)\right]\right)$ (M7)${\;S\text{-}\mathrm{OK}}_{C}=E\left(K_{SC},\;\left[{\mathrm{OK}}_{C}||H^{3}\left(N2\right)\right]\right)$

**Table 3:** Messages M8 to M12 of the KP-SIP protocol.

(M8) $\;\mathrm{Auth}\text{-}\mathrm{CD}2=E(K_{SC},\;[K_{CD1}\left|\left|URI_{C}\right|\right|URI_{D}\left|\left|IP_{S}\right|\right|H^{4}\left(N2\right)\left|\left|N4\right|\right|TS_{4}||LT_{4}])$ (M9) $\;\mathrm{Auth}\text{-}\mathrm{CD}1=E(K_{SD},\;[K_{CD1}\left|\left|URI_{D}\right|\right|URI_{C}\left|\left|IP_{S}\right|\right|H^{3}\left(N3\right)\left|\left|N4\right|\right|TS_{5}||LT_{5}])$ (M10) ${S\text{-}\mathrm{ACK}}_{C}=E\left(K_{CD1},\;\left[\mathrm{ACK}\left|\left|K_{CD2}\right|\right|H\left(N4\right)\right]\right)$ (M11) $S\text{-}\mathrm{BYE}=\left(K_{CD2},\;\left[\mathrm{BYE}||H^{2}\left(N4\right)\left|\left|TS_{6}\right|\right|LT_{6}\right]\right)$ (M12) $S\text{-}\mathrm{OK}\;=\left(K_{CD2},\;\left[\mathrm{OK}||H^{3}\left(N4\right)\right]\right)$

**Table 4:** Comparison of security features.

	F1	F2	F3	F4	F5	F6	F7	F8	F9	F10	F11	F12
[39]	N	Y	Y	Y	N	N	Y	Y	Y	Y	Y	N
[45]	Y	Y	Y	N	N	Y	N	Y	Y	Y	Y	Y
[41]	Y	Y	Y	N	N	Y	N	Y	N	Y	Y	Y
[42]	Y	Y	Y	Y	N	N	Y	Y	N	Y	Y	Y
[43]	Y	Y	Y	Y	N	N	N	Y	Y	Y	Y	Y
[48]	Y	Y	Y	N	N	N	Y	Y	Y	Y	Y	Y
[49]	N	Y	Y	Y	Y	N	Y	Y	Y	Y	Y	Y
[50]	N	Y	Y	Y	Y	N	Y	Y	Y	N	Y	Y
[51]	Y	Y	Y	Y	Y	Y	Y	Y	Y	N	Y	Y
SRP	N	N	N	Y	Y	Y	Y	Y	Y	Y	Y	Y
A-SIP	Y	Y	Y	Y	Y	Y	Y	Y	Y	Y	Y	Y

F1: offline password guessing attack resistance; F2: stolen verifier attack resistance; F3: Denning–Sacco attack resistance; F4: replay attack resistance; F5: user impersonation attack resistance; F6: server impersonation attack resistance; F7: privileged insider attack resistance; F8: MITM attack resistance; F9: providing mutual authentication; F10: providing perfect forward secrecy; F11: providing known key secrecy; F12: session-key temporary information attack resistance. ‘Y’: scheme provides the feature; ‘N’: scheme does not provide the feature.

**Table 5:** Mean and standard deviation for execution time for different cryptographic operations.

Symbol	Operation	Mean (µs)	Stan. Dev. (µs)
$T_{H}$	One-way hash function (SHA-1)	16.5	4.8
$T_{PM}$	Elliptic curve point multiplication	32,646.2	365.5
$T_{PA}$	Elliptic curve point addition	838.7	26.5
$T_{Exp}$	Modular exponentiation	345.4	34.8
$T_{SED}$	Symmetric Key encryption/decryption	157.5	12.8
$T_{R}$	Random number generation	17.4	1.6

**Table 6:** Comparison of the computational cost.

Schemes	Side of Operations	Operations	Cost (ms)	Total (ms)
[39]	Client	$3T_{H}+T_{R}+3T_{PM}+T_{PA}$+$T_{SED}$	99.001	197.620
	Server	$2T_{H}+2T_{R}+3T_{PM}+T_{SED}$	98.619	
[45]	Client	$6T_{H}+T_{R}+4T_{PM}+T_{PA}$	131.539	263.868
	Server	$3T_{H}+2T_{R}+4T_{PM}+2T_{PA}$	132.329	
[41]	Client	$4T_{H}+T_{R}+3T_{PM}$	98.022	196.044
	Server	$4T_{H}+T_{R}+3T_{PM}$	98.022	
[42]	Client	$4T_{H}+T_{R}+2T_{PM}$	65.375	130.767
	Server	$5T_{H}+T_{R}+2T_{PM}$	65.392	
[43]	Client	$4T_{H}+T_{R}+3T_{PM}$	98.022	163.381
	Server	$3T_{H}+T_{R}+2T_{PM}$	65.359	
[48]	Client	$4T_{H}+T_{R}+3T_{PM}+T_{PA}$	98.860	196.882
	Server	$4T_{H}+2T_{R}+3T_{PM}$	98.022	
[49]	Client	$5T_{H}+T_{R}+3T_{PM}+T_{PA}$	98.877	197.265
	Server	$5T_{H}+3T_{R}+3T_{PM}+2T_{SED}$	98.388	
[50]	Client	$5T_{H}+T_{R}+2T_{SED}$	0.572	1.443
	Server	$3T_{H}+2T_{R}+5T_{SED}$	0.871	
[51]	Client	$7T_{H}+2T_{R}+2T_{SED}$	0.465	1.527
	Server	$5T_{H}+2T_{R}+6T_{SED}$	1.062	
SRP	Client	$5T_{H}+T_{R}+3T_{Exp}$	13.136	26.239
	Server	$3T_{H}+T_{R}+3T_{Exp}$	13.103	
A-SIP	Client	$8T_{H}+2T_{R}+6T_{Exp}+T_{SED}$	26.363	44.002
	Server	$7T_{H}+T_{R}+4T_{Exp}+T_{SED}$	17.639	

**Table 7:** *T_A_* and *T_S_* (ms) for protocols SIP, SRP-TLS and S-SIP.

		RTT = 32 ms			RTT = 227 ms		
		SIP	S-SIP	SRP-TLS	SIP	S-SIP	SRP-TLS
*T_A_* (ms)	Mean	33.9	146.6	275.4	229.4	733.1	1389.1
	Stan. Dev.	0.3	2.7	7.3	3.4	9.5	11.4
*T_S_* (ms)	Mean	152.3	171.9	166.9	1032.1	1149.5	1102.8
	Stan. Dev.	2.5	3.6	6.8	12.4	14.8	14.2
Overhead (ms)		NA	132.3	256.1	NA	621.1	1230.4

## Appendix A

ProVerif defines cryptographic primitives as constructors, destructors and equations. Table A1 shows the constructors that we defined to model the proposed protocol. The following shows the main parts of the ProVerif code that are explained in Section 7.

**Table A1:** Constructors defined in the ProVerif model of A-SIP.

Constructor	Function
h	One-way hash function
mult	Multiplication of two integers
mult3	Multiplication of three integers
div	Integer division
concat	Concatenation 2, 3, 4 or 6 values
exp	Exponential operation
xor	Exclusive-OR
sEnc	Symmetric encryption
sDec	Symmetric decryption
gKey	Converting from the bitstring type to the key type
BitKey	Converting from the key type to the bitstring type
getfirst	Extract the first of concatenated values
getsec	Extract the second of concatenated values

$\begin{array}{c} \left(*===\mathrm{channels}====*\right)\\ free\;\mathrm{SCh}:\mathrm{channel}\;\left[private\right].\\ free\;\mathrm{Ch}:\mathrm{channel}.\\ \left(*\;\mathrm{Shared}\;\mathrm{keys}\;*\right)\\ free\;\mathrm{Kc}:\mathrm{key}\;\left[private\right].\\ free\;\mathrm{Ks}:\mathrm{key}\;\left[private\right].\\ free\;\mathrm{Ksc}:\mathrm{key}\;\left[private\right].\\ free\;\mathrm{Kp}:\mathrm{key}\;\left[private\right].\\ \left(*\;\mathrm{constants}\;\&\;\mathrm{Variables}\;*\right)\\ const\;g:\mathrm{bitstring}.\\ const\;p:\mathrm{bitstring}.\\ const\;\mathrm{IDc}:\mathrm{bitstring}.\\ free\;\mathrm{IPs}:\mathrm{bitstring}.\\ free\;\mathrm{Sp}:\mathrm{bitstring}\;\left[private\right].\\ free\;\mathrm{Pwc}:\;\mathrm{bitstring}\;\left[private\right].\\ weaksecret\;\mathrm{PWc}.\\ \left(*====\mathrm{Types}====*\right)\\ type\;\mathrm{key}.\\ \left(*====\mathrm{Functions}====*\right)\\ fun\;h\left(\mathrm{bitstring}\right):\mathrm{bitstring}.\\ fun\;\mathrm{mult}\left(\mathrm{bitstring},\mathrm{bitstring}\right):\mathrm{bitstring}.\\ fun\;\mathrm{mult}3\left(\mathrm{bitstring},\mathrm{bitstring},\mathrm{bitstring}\right):\mathrm{bitstring}.\\ fun\;\mathrm{div}\left(\mathrm{bitstring},\mathrm{bitstring}\right):\mathrm{bitstring}.\\ fun\;\mathrm{concat}\left(\mathrm{bitstring},\mathrm{bitstring}\right):\mathrm{bitstring}.\\ fun\;\mathrm{concat}3\left(\mathrm{bitstring},\mathrm{bitstring},\mathrm{bitstring}\right):\mathrm{bitstring}.\\ fun\;\mathrm{concat}4\left(\mathrm{bitstring},\mathrm{bitstring},\mathrm{bitstring},\;\mathrm{bitstring}\right):\mathrm{bitstring}.\\ fun\;\mathrm{concat}6\left(\mathrm{bitstring},\mathrm{bitstring},\mathrm{bitstring},\mathrm{bitstring},\mathrm{bitstring},\mathrm{bitstring}\right):\mathrm{bitstring}.\\ fun\;\exp\left(\mathrm{bitstring},\mathrm{bitstring}\right):\;\mathrm{bitstring}.\\ fun\;\mathrm{xor}\left(\mathrm{bitstring},\mathrm{bitstring}\right):\;\mathrm{bitstring}.\\ fun\;\mathrm{sEnc}\;\left(\mathrm{key},\mathrm{bitstring}\right):\mathrm{bitstring}.\\ fun\;\mathrm{sDec}\;\left(\mathrm{key},\mathrm{bitstring}\right):\;\mathrm{bitstring}.\\ fun\;\mathrm{gKey}\left(\mathrm{bitstring}\right):\;\mathrm{key}.\\ fun\;\mathrm{BitKey}\left(\mathrm{key}\right):\;\mathrm{bitstring}.\\ fun\;\mathrm{getfirst}\left(\mathrm{bitstring}\right):\;\mathrm{bitstring}.\\ fun\;\mathrm{getsecond}\left(\mathrm{bitstring}\right):\;\mathrm{bitstring}.\\ \left(*========\mathrm{Equations}========*\right)\\ equation\;forall\;x:\mathrm{bitstring},y:\;\mathrm{bitstring};\;\exp\left(\exp\left(g,x\right),y\right)=\exp\left(\exp\left(g,y\right),x\right).\\ equation\;forall\;m:\mathrm{bitstring},k:\mathrm{key};\;\mathrm{sDec}\;\left(k,\mathrm{sEnc}\;\left(k,m\right)\right)=m.\\ equation\;forall\;m:\mathrm{bitstring},k:\mathrm{key};\;\mathrm{sEnc}\;\left(k,\mathrm{sDec}\;\left(k,m\right)\right)=m.\\ equation\;forall\;m:\mathrm{bitstring},k:\mathrm{bitstring};\;\mathrm{xor}\;\left(k,\mathrm{xor}\left(k,m\right)\right)=m.\\ quation\;forall\;m:\mathrm{bitstring},n:\mathrm{bitstring};\;\mathrm{getfirst}\;\left(\mathrm{concat}\left(m,n\right)\right)=m.\\ equation\;forall\;m:\mathrm{bitstring},n:\mathrm{bitstring};\;\mathrm{getsec}\;\left(\mathrm{concat}\left(m,n\right)\right)=n.\\ \left(*====\mathrm{Events}====*\right)\\ event\;\mathrm{UserStarted}\left(\mathrm{bitstring}\right).\\ event\;\mathrm{UserAuthed}\left(\mathrm{bitstring}\right).\\ event\;\mathrm{ServerStarted}\left(\mathrm{bitstring}\right).\\ event\;\mathrm{ServerEnd}\left(\mathrm{bitstring}\right).\\ \left(*=====\mathrm{Main}=====*\right)\\ process\;\left(!ProcessServer|!ProcessClient\right)\\ \left(*=====\mathrm{Queries}=====*\right)\\ query\;attacker\left(\mathrm{Kc}\right).\\ query\;attacker\left(\mathrm{Ks}\right).\\ query\;attacker\left(\mathrm{Ksc}\right).\\ query\;attacker\left(\mathrm{Kp}\right)\\ query\;id:\mathrm{bitstring};\;inj\text{-}event\left(\mathrm{UserAuthed}\left(id\right)\right)\;\Rightarrow inj\text{-}event\left(\mathrm{UserStarted}\left(id\right)\right).\\ query\;id:\mathrm{bitstring};\;inj\text{-}event\left(\mathrm{ServerEnd}\left(id\right)\right)\;\Rightarrow inj\text{-}event\left(\mathrm{ServerStarted}\left(id\right)\right). \end{array}$
